# Supplementary material for: Mutation of key signaling regulators of cerebrovascular development in vein of Galen malformations
Source: Nat Commun. 2023 Nov 17;14:7452. doi: 10.1038/s41467-023-43062-z (PMC10656524; doi:10.1038/s41467-023-43062-z)
Supplement: Supplementary file 1 — Supplementary Information File [file 41467_2023_43062_MOESM1_ESM.pdf]

**Mutation of key signaling regulators of cerebrovascular development in vein of Galen malformations**

Shujuan Zhao[1,2†], Kedous Y. Mekbib[2,3†], Martijn A. van der Ent[4†], Garrett Allington[2,5†], Andrew Prendergast[6], Jocelyn E. Chau[7], Hannah Smith[2,3], John Shohfi[2,3], Jack Ocken[3], Daniel Duran[8], Charuta G. Furey[3], Hao Thi Le[2], Phan Q. Duy[3], Benjamin C. Reeves[3], Junhui Zhang[9], Carol Nelson-Williams[9], Di Chen[4], Boyang Li[10], Timothy Nottoli[11], Suxia Bai[11], Myron Rolle[3], Xue Zeng[7,12], Weilai Dong[9,12], Po-Ying Fu[1], Yung-Chun Wang[1], Shrikant Mane[9], Paulina Piwowarczyk[13], Katie Pricola Fehnel[13], Alfred Pokmeng See[13], Bermans J. Iskandar[14], Beverly Aagaard-Kienitz[14,15], Quentin J. Moyer[2], Evan Dennis[2], Emre Kiziltug[2], Adam J. Kundishora[3], Tyrone DeSpenza Jr.[3], Ana B.W. Greenberg[2], Seblewengel M. Kidanemariam[16], Andrew T. Hale[17], James M. Johnston[17], Eric M.Jackson[18], Phillip B. Storm[19,20], Shih-Shan Lang[19,20], William E. Butler[2], Bob S. Carter[2], Paul Chapman[2], Christopher J. Stapleton[2], Aman B. Patel[2], Georges Rodesch[21], Stanislas Smajda[21], Alejandro Berenstein[22], Tanyeri Barak[3], E. Zeynep Erson-Omay[3], Hongyu Zhao[9,10], Andres Moreno-De-Luca[23], Mark R. Proctor[13], Edward R. Smith[13], Darren B. Orbach[13,24], Seth L. Alper[25], Stefania Nicoli[9,26,27], Titus J. Boggon[7,28], Richard P. Lifton[12], Murat Gunel[3], Philip D. King[4#†], Sheng Chih Jin[1,29#†], and Kristopher T. Kahle[2,3,30,31#†]

## Contents:

**Supplementary Figures 1-11:**
Supplementary Figure 1. Representative imaging for probands.
Supplementary Figure 2. Variants in *RASA1* and *EPHB4.*
Supplementary Figure 3. Steady-state abundance of EPHB4 D-mis mutants.
Supplementary Figure 4. Variants in *NOTCH1*, *PTPN11* and *ITGB1*.
Supplementary Figure 5. *De novo* mutation rate closely approximates Poisson distribution in VOGM cases and controls.
Supplementary Figure 6. Cutaneous manifestations in VOGM probands and family members.
Supplementary Figure 7. GO term and pathway enrichment analysis.
Supplementary Figure 8. Quantification of vessel parameters following *ephb4a/b* loss of function.
Supplementary Figure 9. Quantification of vessel parameters following acvrl1 loss of function.
Supplementary Figure 10. The remaining top 10 GO biological processes, molecular functions, cellular components, and GO WikiPathways enrichment terms.
Supplementary Figure 11. VOGM gene discovery projections.

**Supplementary Tables 1-15:**
Supplementary Table 1. VOGM patient clinical and demographic characteristics.
Supplementary Table 2. Summary sequencing statistics for the VOGM case and control cohorts.
Supplementary Table 3. *De novo* variant enrichment analysis for each mutational class in 90 VOGM cases and controls.
Supplementary Table 4. DenovolyzeR output of top 20 significant genes
Supplementary Table 5. Top 20 significant genes in binomial test of damaging variants enrichment.
Supplementary Table 6. Top 20 significant genes in case-control burden test of damaging variants.
Supplementary Table 7. Phenotypes of probands with variants in *RASA1* and *EPHB4.*
Supplementary Table 8. Top 10 term of gene ontologies and pathway analysis.
Supplementary Table 9. Damaging variants in axon guidance genes.
Supplementary Table 10. Characteristics of patients with *de novo* and transmitted variant in other interesting genes.
Supplementary Table 11. Summary of clinical features of patients with *de novo* and transmitted variant in other interesting genes.
Supplementary Table 12. Damaging variants in Ras signaling pathway.
Supplementary Table 13. Module gene sets.
Supplementary Table 14. Damaging recessive mutations in 114 VOGM cases.
Supplementary Table 15. Gene-specific crRNA sequences designed using CRISPR.

**References**

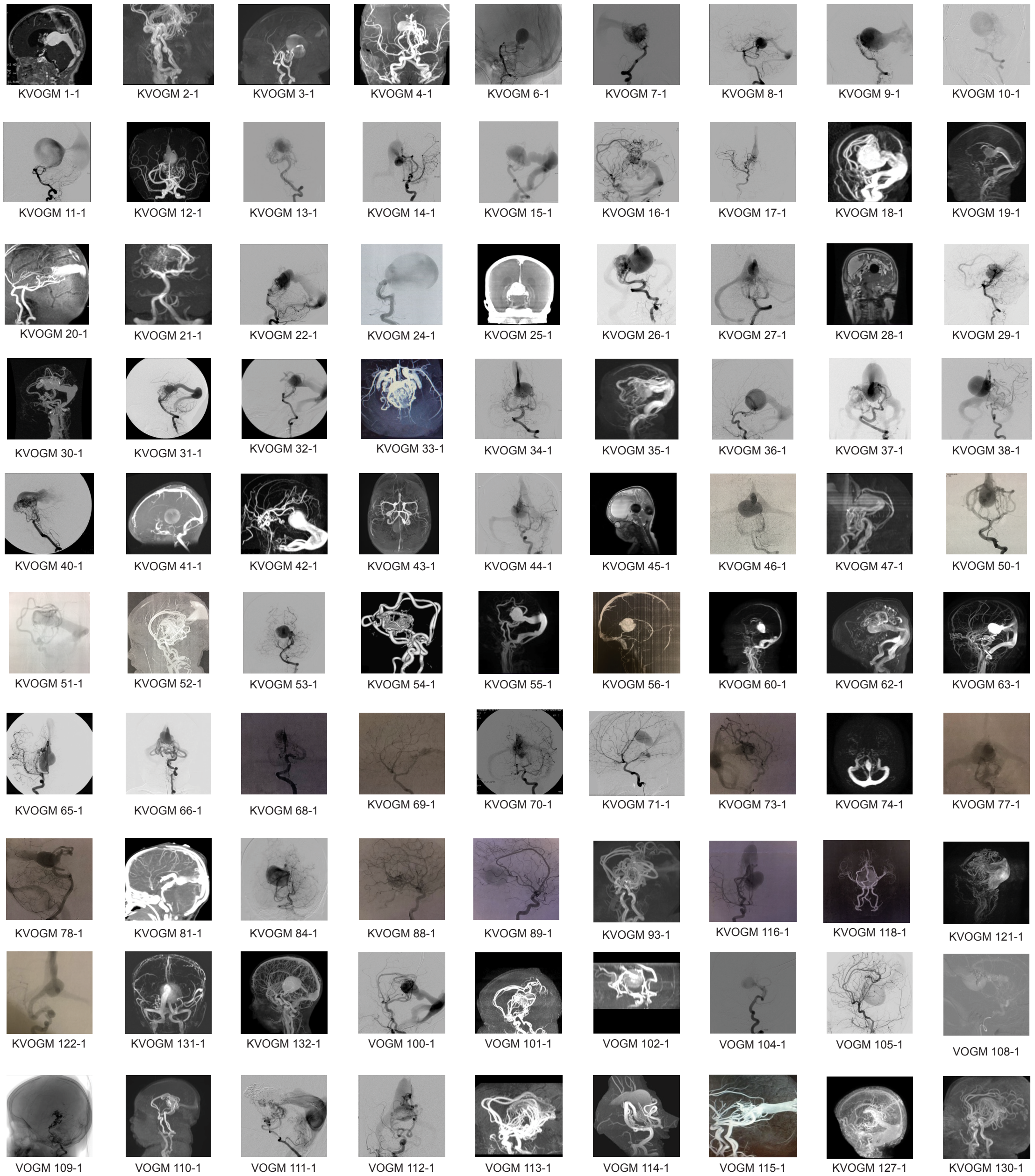


**Supplementary Figure 1. Representative imaging for probands.** Representative images of 3-Tesla time-of-flight magnetic resonance angiography or digital subtraction angiography for all patients with available imaging, with patient codes.

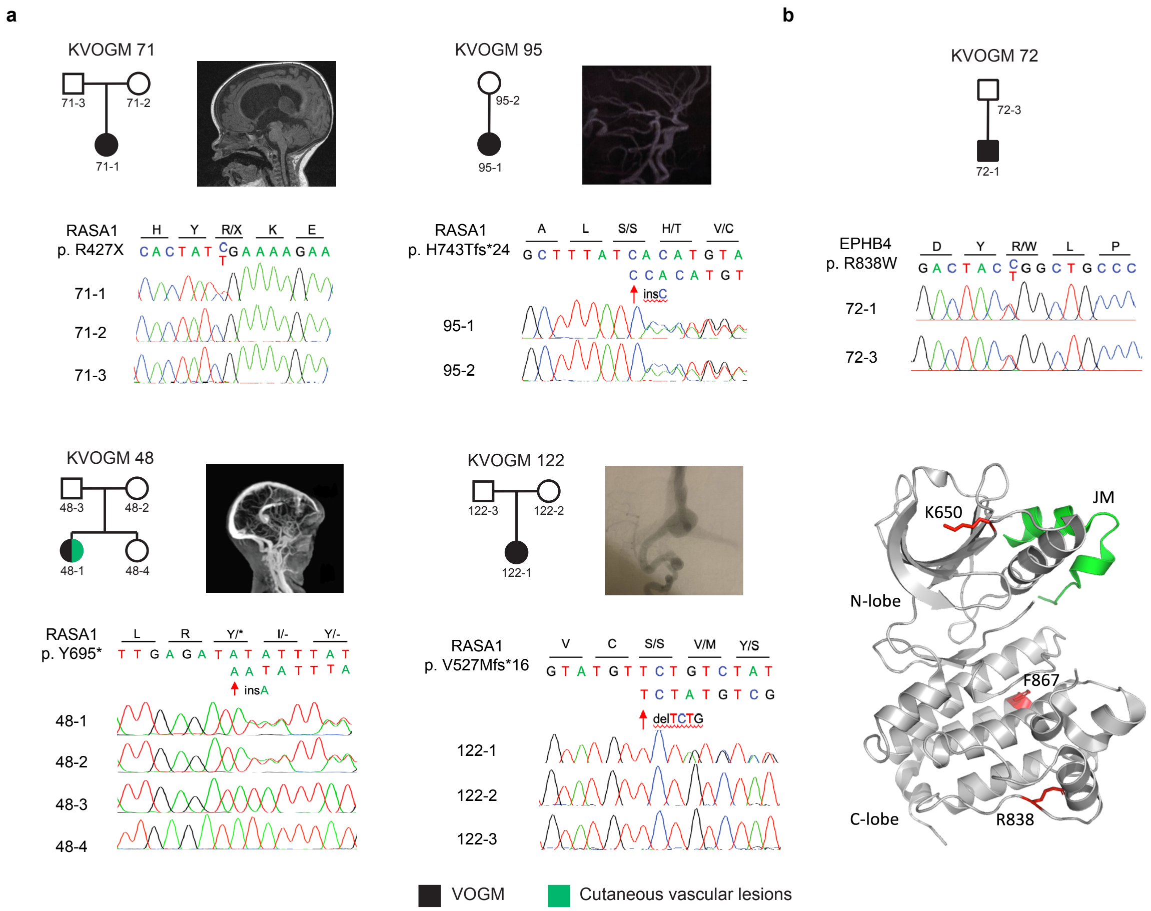


**Supplementary Figure 2. Variants in *RASA1* and *EPHB4*.** (A) *De novo* and transmitted variants in *RASA1*. Representative digital subtraction angiography reconstructions and pedigrees depicting kindred structure and phenotype. Black symbol represents vein of Galen malformation (VOGM) and green symbols denote cutaneous vascular lesions. These variants (p. R427X, p. V527Mfs*16, p. Y695* and p. H743Tfs*24) are confirmed by direct PCR amplification with custom primers followed by Sanger sequencing. For Sanger sequence of variant p. R709X, please refer to our previous publication[1]. (B) Transmitted variants in *EPHB4*. Representative digital subtraction angiography reconstructions and pedigree depicting kindred structure and phenotype of KVOGM-72. Variant (p. R838W) was validated by Sanger sequencing. Ribbon structure of EphB4 kinase domain (PDB ID: 6FNL) [2] in grey with juxtamembrane region (JM) from an aligned EphB2 structure (PDB ID: 1JPA) [3] in green. Locations of variants p. K650, p. R838 and p. F867 are indicated in red. For Sanger sequences of variants p. F867L, p. K650N, p. A509G, p. E432Gfs*7, please refer to our previous publication[1].

**a**

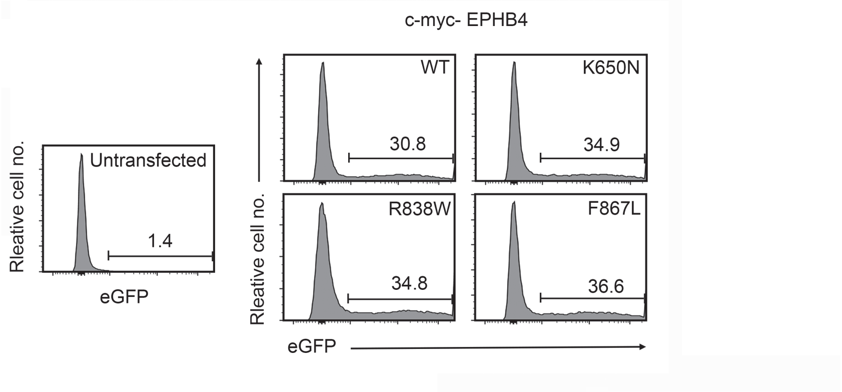


**b**

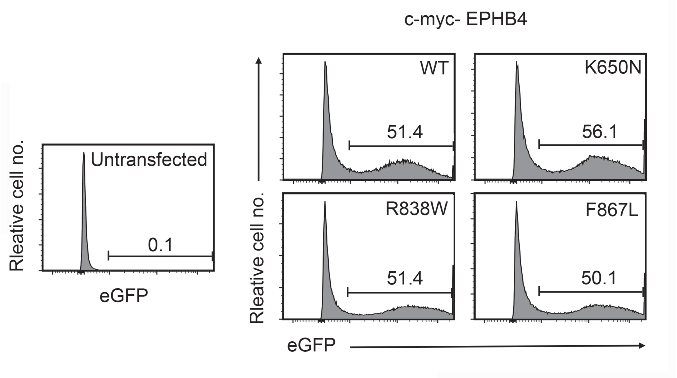


**Supplementary Figure 3. Steady-state abundance of EPHB4 D-mis mutants**. Cos-7 cells were transiently transfected with c-myc-tagged WT or EPHB4 D-mis mutants together with an eGFP-encoding vector to assess transfection efficiency by flow cytometry. Numbers indicate % of eGFP+ cells for separate experiments corresponding to left and right parts of Figure 1E.

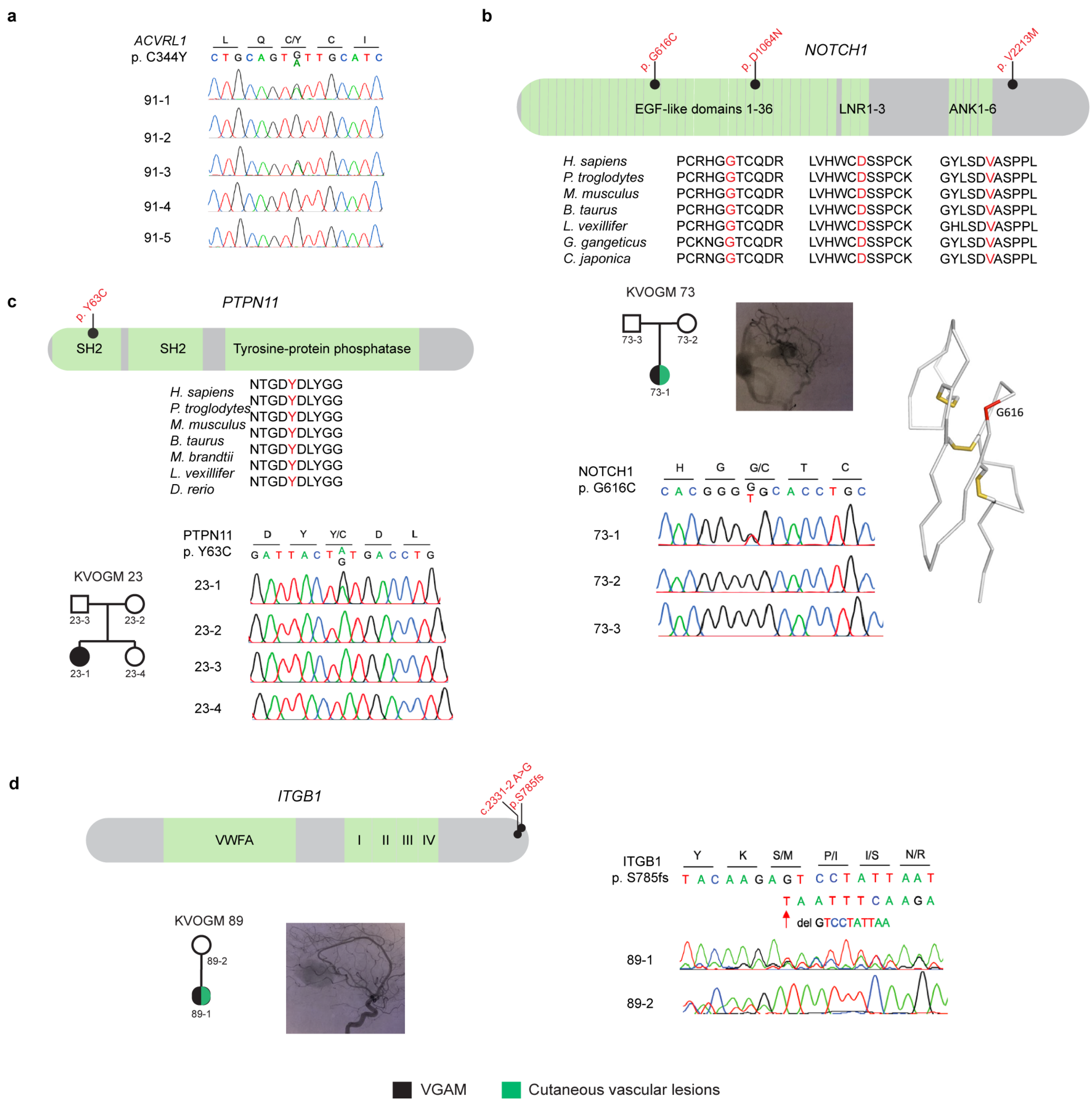


**Supplementary Figure 4. Variants in *NOTCH1*, *PTPN11* and *ITGB1*.** (A) Multi-generational VOGM family in KVOGM-91 with Sanger-validated genotype. See Figure 2C for pedigree information of this family. (B) Transmitted and de novo variant in *NOTCH1*. Transmitted variant p.V2213M in VGOM46-1, unphased variant p.D1064N in KVOGM83-1, and DNV p.G616C in KVOGM73-1 mapped to protein domains. Representative digital subtraction angiography reconstruction and pedigree depicting kindred structure and phenotype for KVOGM73 harboring Sanger-validated *de novo* variant encoding mutant G616C. Schematic illustrating position of G616 in Alphafold model of this domain (AF-P46531-F1-model_v2.pdb, residues 603-641) in the 16th EGF domain of NOTCH1. Disulfides are yellow and G616 is red. (C) *De novo* variant in *PTPN11* mapping to the SH2 domain. KVOGM23 pedigree depicting kindred structure and phenotype. Sanger-validated gene sequence encoding variant p. Y63C. SH2, Src homology 2 domain. (D) Transmitted variants in *ITGB1* mapped to their protein domains. Representative digital subtraction angiography reconstruction and pedigree depicting kindred structure and phenotype. Sanger-validated gene sequence encoding variant p. S785fs.

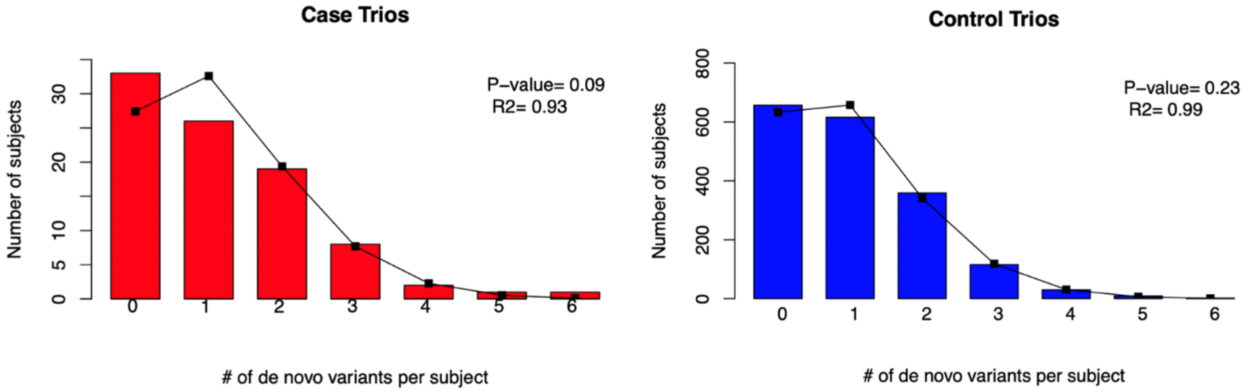


**Supplementary Figure 5*. De novo* mutation rate closely approximates Poisson distribution in VOGM cases and controls.** The observed number of *de novo* variants per subject (bars) is compared to the numbers expected (line) from the Poisson distribution in the case (red) and control cohorts (blue). 'p' denotes chi-squared p-value.

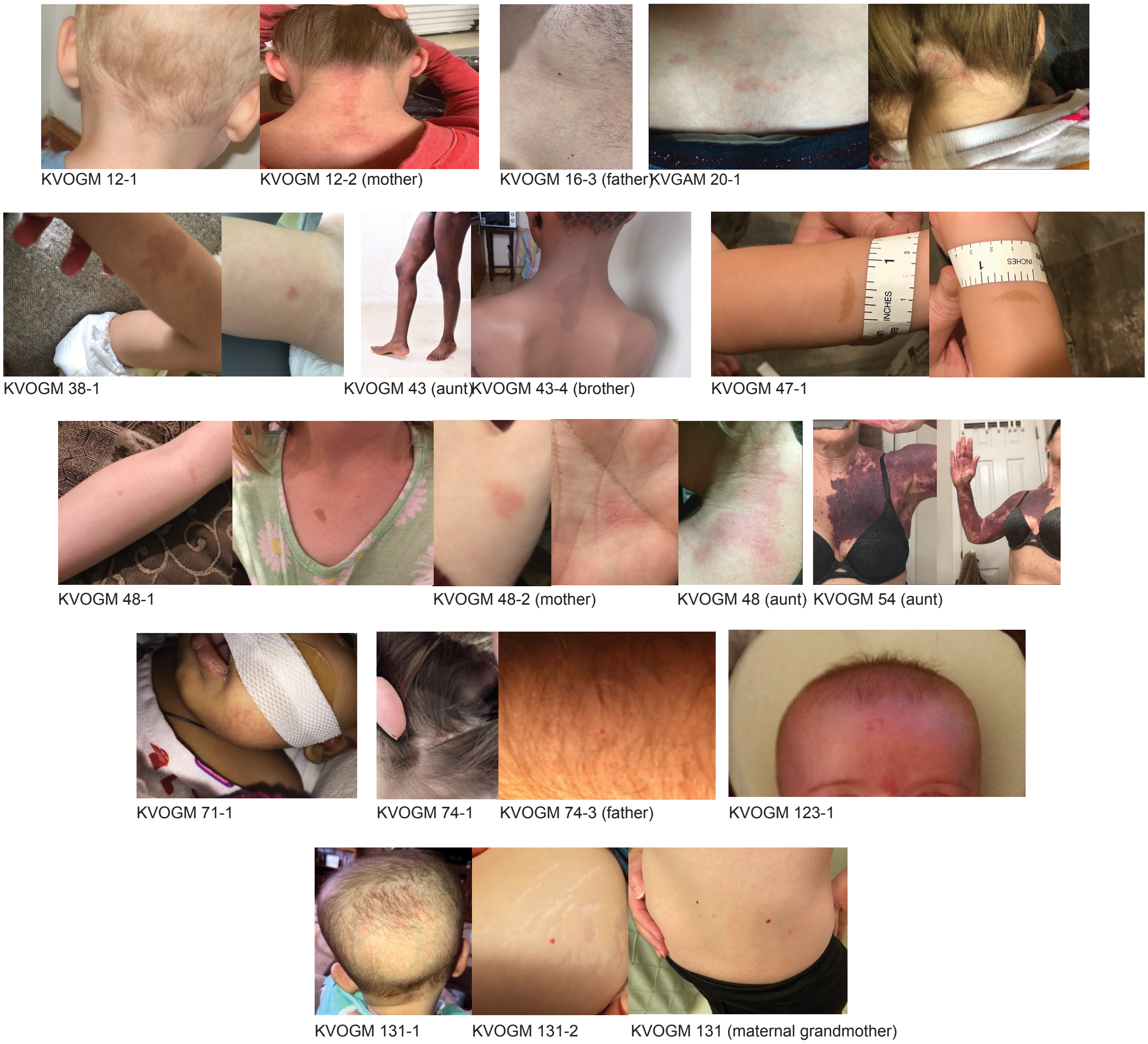


**Supplementary Figure 6. Cutaneous manifestations in VOGM probands and family members.** Atypical capillary malformations and other cutaneous vascular lesions in probands and family members, labelled by patient code and familial relation.

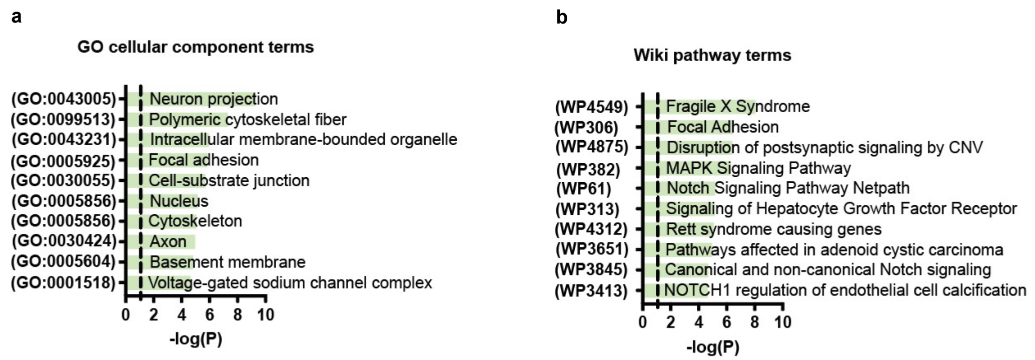


**Supplementary Figure 7. GO term and pathway enrichment analysis.** (A) Top 10 GO cellular component and (B) GO Wiki pathway enrichment terms. The y-axis depicts GO term or WikiPathways term ID numbers. The x axis depicts -log (P value) and the dotted line represents the $\alpha$ = 0.05 significance threshold. The GO term and WikiPathways term name overline their respective bars.

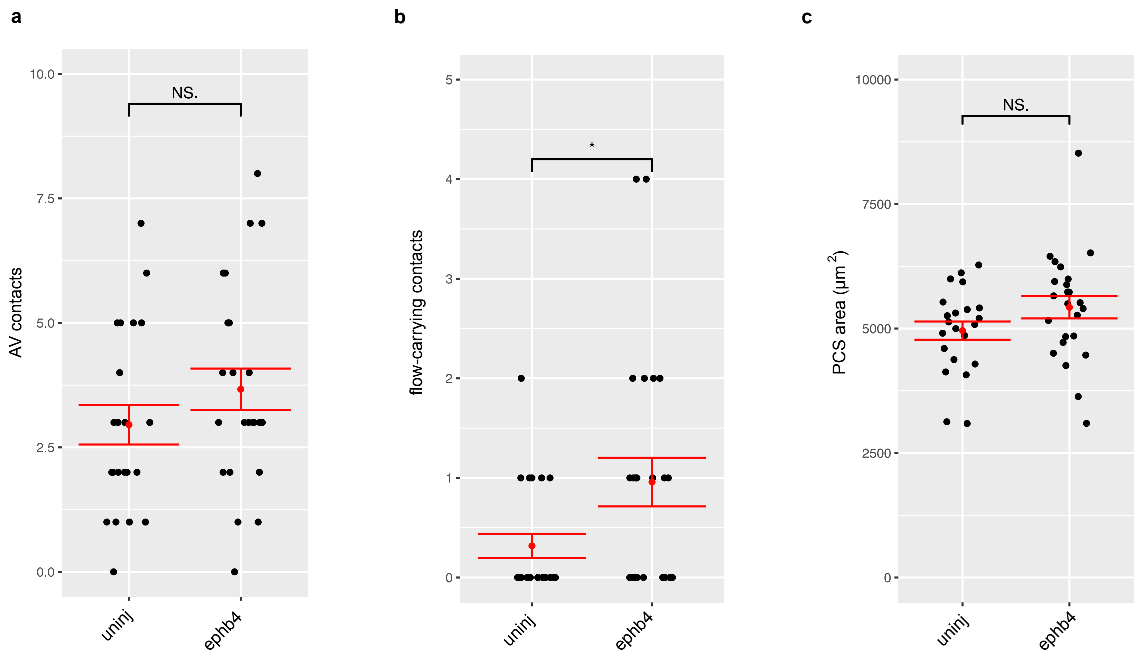


**Supplementary Figure 8. Quantification of vessel parameters following *ephb4a/b* loss of function.** (A) Abnormal contacts between BA and PHBC comparing uninjected and *ephb4a/b* gRNA injected fish. (B) Abnormal flow-carrying contacts between BA and PHBC. (C) PCS area. *** ≤ 0.001, ** ≤ 0.01, * ≤ 0.05. *n* = 46 independent larvae taken from 15 independent experiments. Error bars are ± standard error of the mean.

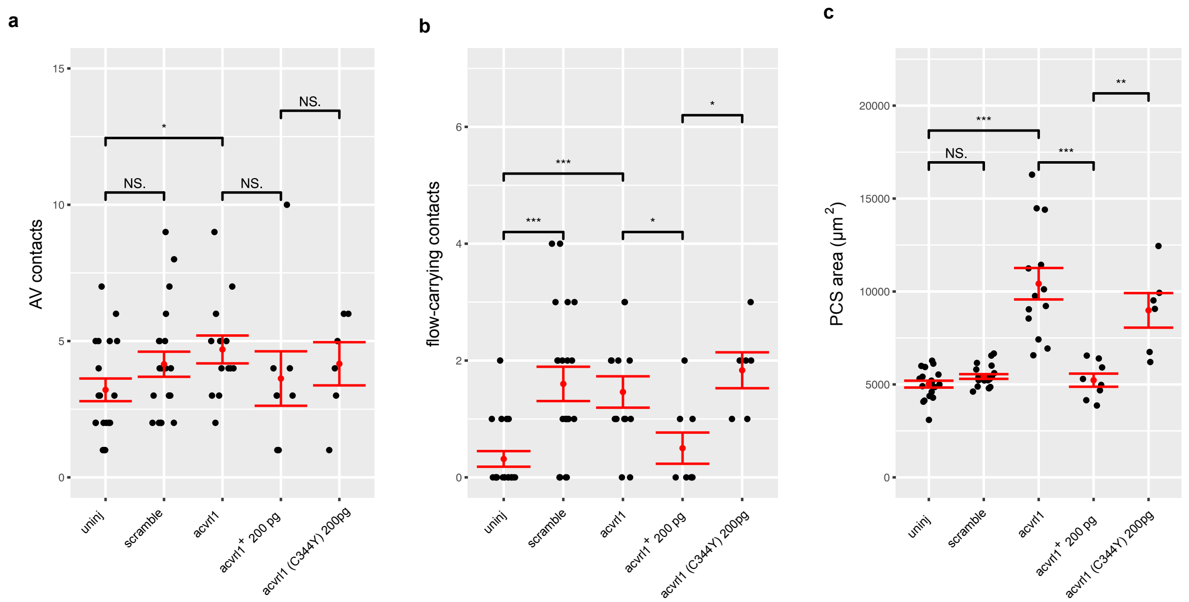


**Supplementary Figure 9. Quantification of vessel parameters following acvrl1 loss of function.** (A) Abnormal contacts between BA and PHBC comparing uninjected, *acvrl1* scramble gRNA, *acvrl1* gRNA, 200 pg *wt acvrl1* rescue, and 200 pg C344Y mutant *acvrl1* false rescue. (B) Abnormal flow-carrying contacts between BA and PHBC. (C) PCS area. *** ≤ 0.001, ** ≤ 0.01, * ≤ 0.05. *n* = 66 independent larvae taken from 14 independent experiments. Error bars are ± standard error of the mean.

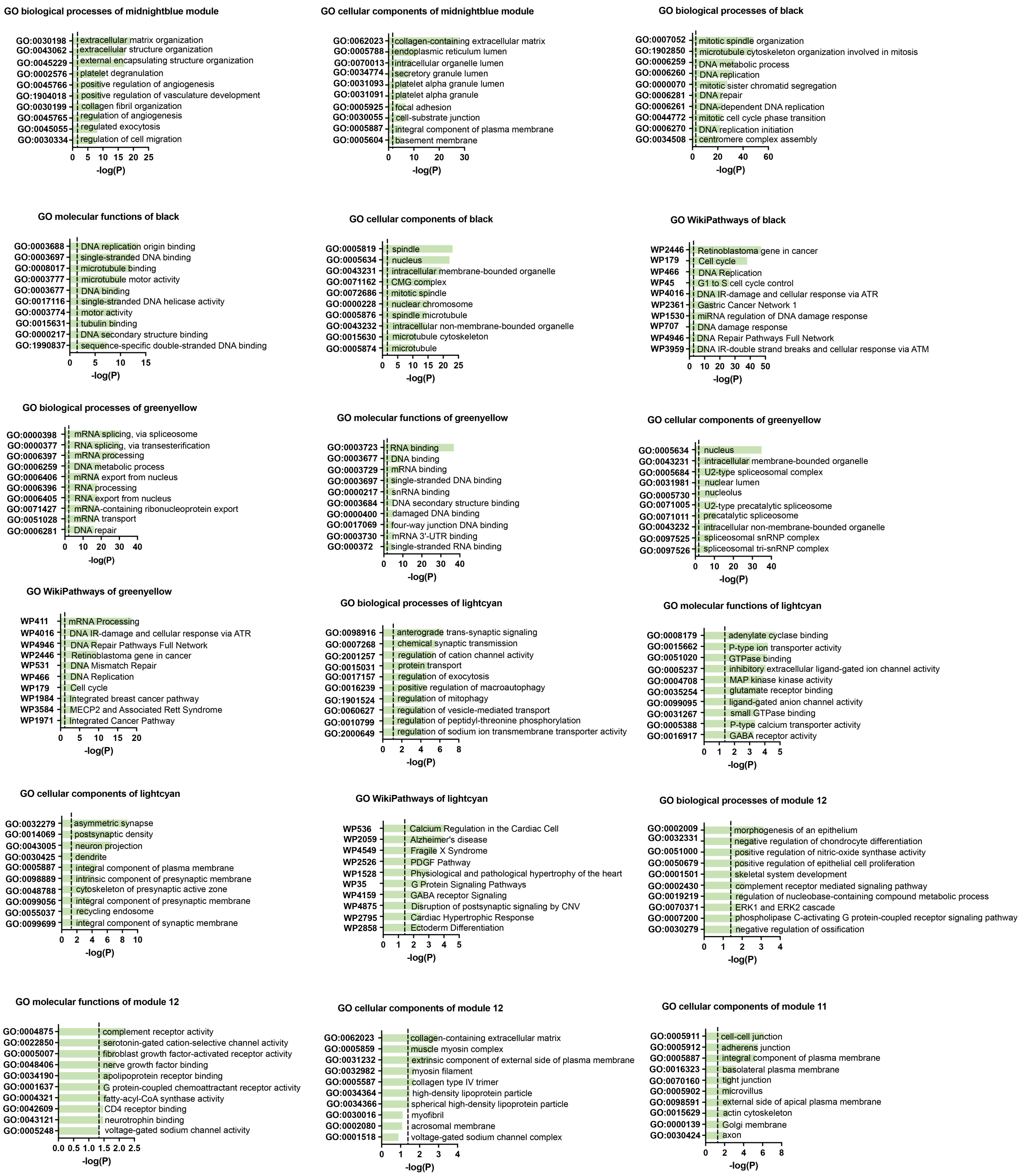


**Supplementary Figure 10**. **The remaining top 10 GO biological processes, molecular functions, cellular components, and GO WikiPathways enrichment terms.** Enrichment analysis for modules in the midgestational human cortex, including the "Midnight Blue" module, the "Black" module, "Green-yellow" module, and the "Light Cyan" module. Enriched terms in sc-RNAseq transcriptomic atlas of human cerebrovasculature in Modules 11 and 12 are also shown. The y-axis depicts GO term or WikiPathways term ID numbers. The x axis depicts -log (P value) and the dotted line represents the $\alpha$ = 0.05 significance threshold. The GO term and WikiPathways term name overline their respective bars.

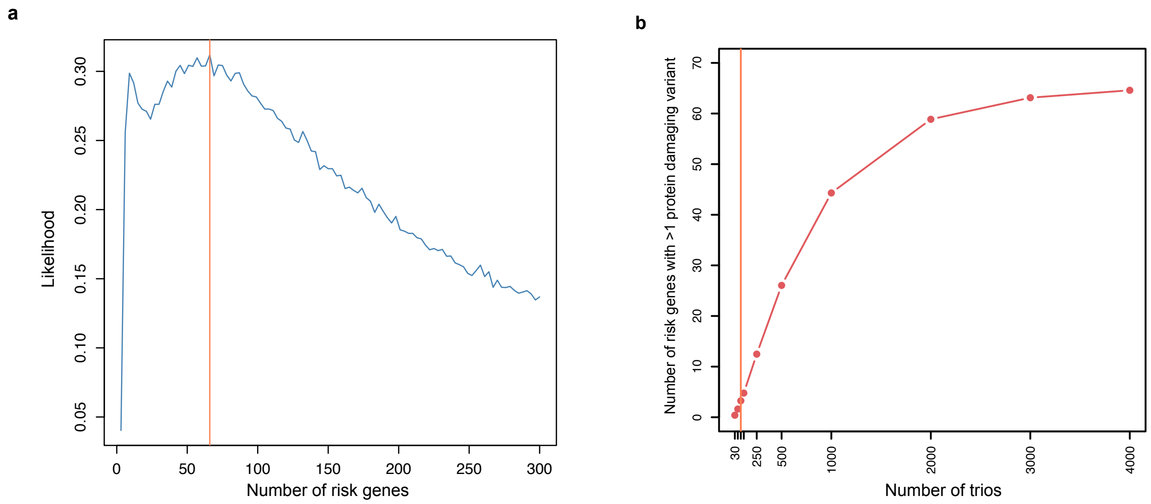


**Supplementary Figure 11. VOGM gene discovery projections.** (A) Estimated number of VOGM risk genes via a *de novo* mechanism. A Monte Carlo simulation was performed based on observed protein-altering *de novo* variants in 3,049 loss-of-function intolerant genes (pLI ≥ 0.9 in gnomAD [v2.1.1]) using 20,000 iterations. We estimate the number of risk genes via a *de novo* mechanism is ~66. (B) Estimated number of genes with more than one protein-damaging variant with an increasing number of trios. The number of trios is specified on the x-axis and the number of genes with more than one protein-damaging *de novo* variant on the y-axis. The expected rate of protein-altering *de novo* variants was modeled in 10,000 iterations with an increasing number of trios, given the probability of *de novo* protein-altering variants. WES of 250 and 1,000 trios are expected to yield a saturation rate of 20.1% and 70.5% respectively, for all VOGM risk genes.

**Supplementary Table 1. VOGM patient clinical and demographic characteristics.**

| Gender | | | Number(percentage) |
|---|---|---|---|
| | Female | | 39 (34.21%) |
| | Male | | 70 (61.40%) |
| | Unknown | | 5 (4.39%) |
| | | Total | 114 (100.00%) |
| Age at diagnosis | | | |
| | Prenatal | | 39 (34.21%) |
| | | Neonate (0-30 days) | 23 (20.18%) |
| | | Infant (1 month-2 years) | 32 (28.07%) |
| | Postnatal | Young child (3-6 years) | 3 (2.63%) |
| | | Child (7-12 years) | 1 (0.88%) |
| | | Adolescent (13-17 years) | 1 (0.88%) |
| | Unknown | | 54 (47.37%) |
| | | Total | 114 (100.00%) |
| Term vs. preterm delivery | | | |
| | Term (≥37 wks) | | 87 (76.32%) |
| | Preterm | | 14 (12.28%) |
| | Unknown | | 13 (11.40%) |
| | | Total | 114 (100.00%) |
| Family history of cutaneous vascular abnormalities* | | | |
| | Yes | | 48 (42.11%) |
| | No | | 45 (39.47%) |
| | Unknown | | 21 (18.42%) |
| | | Total | 114 (100.00%) |
| Self-reported race | | | |
| | African | | 6 (5.26%) |
| | Asian | | 8 (7.02%) |
| | Caucasian | | 73 (64.04%) |
| | Hispanic | | 1 (0.88%) |
| | Latin American | | 0 (0.00%) |
| | Native American | | 0 (0.00%) |
| | Mixed Race | | 10 (8.77%) |
| | Other | | 1 (0.88%) |
| | Unknown | | 15 (13.16%) |
| | | Total | 114 (100.00%) |
| Associated conditions | | | |
| | High output heart failure | | 46 (40.35%) |
| | Progressive macrocephaly | | 55 (48.25%) |
| | Developmental delays | | 62 (54.39%) |
| | Hydrocephalus | | 55 (48.25%) |
| | Headaches | | 25 (21.93%) |
| | Intracranial hemorrhage | | 24 (21.05%) |
| | Prominent veins on face and scalp | | 51 (44.74%) |
| | Recurrent nosebleeds | | 5 (4.39%) |
| | Renal/kidney disease | | 3 (2.63%) |
| | Seizures | | 36 (31.58%) |
| | Cutaneous vascular lesions | | 25 (21.93%) |

*History of vascular birthmarks was self-reported in most cases, including first and second-degree relatives.

**Supplementary Table 2. Summary sequencing statistics for the VOGM case and control cohorts.**

| Category | Cases (MedExome; N=76)* | Cases (xGen IDT; N=234) | Controls (Roche V2; N=5,394) |
|---|---|---|---|
| Read length (bp) | 101 | 101 | 50-99 |
| # of reads per sample (M) | 63.6 | 62.9 | 111.5 |
| Median coverage at each targeted base (X) | 61.3 | 61.1 | 68.0 |
| Mean coverage at each targeted base (X) | 66.3 | 66.1 | 80.8 |
| % of all reads that map to target | 56.3% | 56.6% | 46.5% |
| % of all bases that map to target | 43.1% | 43.3% | 35.7% |
| % of targeted bases read at least 8x | 97.5% | 97.6% | 92.7% |
| % of targeted bases read at least 10x | 96.9% | 97.0% | 91.4% |
| % of targeted bases read at least 15x | 94.4% | 94.5% | 87.8% |

*76 case samples were sequenced using the MedExome capture reagent. All other samples were sequenced using the xGEN Exome Research Panel v1.0 capture reagent (IDT). 8X, 10X and 15X were comparable across the platforms.

**Supplementary Table 3. *De novo* variant enrichment analysis for each mutational class in 90 VOGM cases and controls.**

| VOGM cases, N = 90 | | | | | | | Controls, N=1,798 | | | | | | |
|---|---|---|---|---|---|---|---|---|---|---|---|---|---|
| | Observed | | Expected | | Enrichment | p | | Observed | | Expected | | Enrichment | p |
| | N | Rate | N | Rate | | | | N | Rate | N | Rate | | |
| All genes (N=19,347) | | | | | | | All genes (N=19,347) | | | | | | |
| Total | 107 | 1.19 | 99.7 | 1.11 | 1.07 | 0.25 | Total | 1839 | 1.02 | 1977.1 | 1.10 | 0.93 | 1.00 |
| Syn | 28 | 0.31 | 28.2 | 0.31 | 0.99 | 0.54 | Syn | 492 | 0.27 | 559.8 | 0.31 | 0.88 | 1.00 |
| T-Mis | 46 | 0.51 | 49.4 | 0.55 | 0.93 | 0.71 | T-Mis | 949 | 0.53 | 979.3 | 0.54 | 0.97 | 0.84 |
| D-Mis | 22 | 0.24 | 13.4 | 0.15 | 1.65 | 0.02 | D-Mis | 248 | 0.14 | 266.7 | 0.15 | 0.93 | 0.88 |
| LoF | 11 | 0.12 | 8.7 | 0.1 | 1.27 | 0.26 | LoF | 150 | 0.08 | 171.3 | 0.10 | 0.88 | 0.95 |
| Protein-altering | 79 | 0.88 | 71.5 | 0.79 | 1.11 | 0.20 | Protein-altering | 1347 | 0.75 | 1417.3 | 0.79 | 0.95 | 0.97 |
| Protein-damaging | 33 | 0.37 | 22 | 0.24 | 1.5 | 0.02 | Protein-damaging | 398 | 0.22 | 438 | 0.24 | 0.91 | 0.98 |
| High brain-expressed genes (brain expression rank ≥ 75%; n=4,522) | | | | | | | High brain-expressed genes (brain expression rank ≥ 75%; n=4,522) | | | | | | |
| Total | 27 | 0.3 | 25.7 | 0.29 | 1.05 | 0.43 | Total | 515 | 0.29 | 511.6 | 0.28 | 1.01 | 0.45 |
| Syn | 8 | 0.09 | 7.2 | 0.08 | 1.11 | 0.43 | Syn | 128 | 0.07 | 143 | 0.08 | 0.9 | 0.9 |
| T-Mis | 9 | 0.1 | 12.2 | 0.14 | 0.74 | 0.86 | T-Mis | 274 | 0.15 | 243.6 | 0.14 | 1.12 | 0.03 |
| D-Mis | 4 | 0.04 | 4 | 0.04 | 1.01 | 0.56 | D-Mis | 74 | 0.04 | 78.9 | 0.04 | 0.94 | 0.72 |
| LoF | 6 | 0.07 | 2.3 | 0.03 | 2.58 | 0.03 | LoF | 39 | 0.02 | 46.2 | 0.03 | 0.85 | 0.87 |
| Protein-altering | 19 | 0.21 | 18.5 | 0.21 | 1.03 | 0.49 | Protein-altering | 387 | 0.22 | 368.6 | 0.21 | 1.05 | 0.18 |
| Protein-damaging | 10 | 0.11 | 6.3 | 0.07 | 1.59 | 0.10 | Protein-damaging | 113 | 0.06 | 125 | 0.07 | 0.9 | 0.87 |
| Loss-of-function intolerant genes (gnomADv2.1.1 pLI ≥ 0.9; n=3,049) | | | | | | | Loss-of-function intolerant genes (gnomADv2.1.1 pLI ≥ 0.9; n=3,049) | | | | | | |
| Total | 22 | 0.24 | 23.9 | 0.27 | 0.92 | 0.68 | Total | 456 | 0.25 | 473.5 | 0.26 | 0.96 | 0.83 |
| Syn | 4 | 0.04 | 6.7 | 0.07 | 0.59 | 0.90 | Syn | 115 | 0.06 | 133.4 | 0.07 | 0.86 | 0.96 |
| T-Mis | 6 | 0.07 | 10.9 | 0.12 | 0.55 | 0.96 | Mis | 233 | 0.13 | 213.5 | 0.12 | 1.08 | 0.12 |
| D-Mis | 9 | 0.1 | 4.2 | 0.05 | 2.12 | 0.03 | D-Mis | 75 | 0.04 | 84.5 | 0.05 | 0.88 | 0.87 |
| LoF | 3 | 0.03 | 2.1 | 0.02 | 1.4 | 0.36 | LoF | 33 | 0.02 | 42.2 | 0.02 | 0.78 | 0.94 |
| Protein-altering | 18 | 0.2 | 17.2 | 0.19 | 1.05 | 0.45 | Protein-altering | 341 | 0.19 | 340.2 | 0.19 | 1 | 0.53 |
| Protein-damaging | 12 | 0.13 | 6.4 | 0.07 | 1.88 | 0.03 | Protein-damaging | 108 | 0.06 | 126.7 | 0.07 | 0.85 | 0.96 |
| Loss-of-function-intolerant & high brain expressed genes (n=1,495) | | | | | | | Loss-of-function-intolerant & high brain expressed genes (n=1,495) | | | | | | |
| Total | 12 | 0.13 | 11.4 | 0.13 | 1.05 | 0.47 | Total | 228 | 0.13 | 227.9 | 0.13 | 1 | 0.51 |
| Syn | 2 | 0.02 | 3.2 | 0.04 | 0.63 | 0.83 | Syn | 58 | 0.03 | 63.3 | 0.04 | 0.92 | 0.76 |
| T-Mis | 3 | 0.03 | 5.1 | 0.06 | 0.59 | 0.88 | Mis | 112 | 0.06 | 102.2 | 0.06 | 1.1 | 0.18 |
| D-Mis | 4 | 0.04 | 2.1 | 0.02 | 1.93 | 0.16 | D-Mis | 41 | 0.02 | 41.5 | 0.02 | 0.99 | 0.55 |
| LoF | 3 | 0.03 | 1.1 | 0.01 | 2.85 | 0.09 | LoF | 17 | 0.01 | 20.9 | 0.01 | 0.81 | 0.83 |
| Protein-altering | 10 | 0.11 | 8.3 | 0.09 | 1.21 | 0.32 | Protein-altering | 170 | 0.1 | 164.6 | 0.09 | 1.03 | 0.35 |
| Protein-damaging | 7 | 0.08 | 3.1 | 0.03 | 2.24 | 0.04 | Protein-damaging | 58 | 0.03 | 62.4 | 0.03 | 0.93 | 0.73 |

N: number of de novo variants (DNVs); Rate: number of DNVs per subject; Enrichment: ratio of observed to expected numbers of DNVs; D-mis: damaging missense variants as predicted by MetaSVM or MPC >2; T-mis: tolerated missense variants as predicted by MetaSVM or MPC <2; LoF: loss-of-function variants comprised of premature termination, frameshift, or splice-site variants; Significance threshold determined by upper cumulative quantile (one-sided) of Poisson distribution. Not adjusted.

**Supplementary Table 4. DenovolyzeR output of top 20 significant genes.**

| Gene | Observed | Expected | fold-change | min_pValue |
|---|---|---|---|---|
| *RASA1* | 2 | 9.79E-04 | 2042.48 | 4.79E-07 |
| *KEL* | 2 | 4.54E-03 | 440.74 | 1.03E-05 |
| *SLC35E4* | 1 | 3.63E-04 | 2754.37 | 3.63E-04 |
| *ZSWIM2* | 1 | 8.35E-04 | 1197.32 | 8.35E-04 |
| *SAT1* | 1 | 4.42E-04 | 2264.80 | 4.41E-04 |
| *CYP3A5* | 1 | 3.76E-04 | 2658.16 | 3.76E-04 |
| *RAB11FIP4* | 1 | 4.48E-04 | 2231.15 | 4.48E-04 |
| *TCAP* | 1 | 6.08E-04 | 1643.66 | 6.08E-04 |
| *WDR76* | 1 | 5.29E-04 | 1889.64 | 5.29E-04 |
| *SIRT1* | 1 | 5.78E-04 | 1730.70 | 5.78E-04 |
| *PDCD5* | 1 | 6.71E-04 | 1491.02 | 6.70E-04 |
| *ZFAT* | 1 | 7.24E-04 | 1381.98 | 7.23E-04 |
| *LPGAT1* | 1 | 1.15E-03 | 872.14 | 1.15E-03 |
| *ALG12* | 1 | 1.28E-03 | 781.37 | 1.28E-03 |
| *TMEM128* | 1 | 1.05E-03 | 951.29 | 1.05E-03 |
| *PLA2G5* | 1 | 1.08E-03 | 925.46 | 1.08E-03 |
| *PLXNB1* | 1 | 1.30E-03 | 770.53 | 1.30E-03 |
| *SLC6A12* | 1 | 1.93E-03 | 518.73 | 1.93E-03 |
| *KCNJ10* | 1 | 1.78E-03 | 562.87 | 1.78E-03 |
| *FLNB* | 1 | 1.59E-03 | 628.46 | 1.59E-03 |

RASA1 exhibits exome-wide significant enrichment for all rare variants in VOGM cases. Rows above the double line indicate those genes that surpass exome-wide significance threshold. The exome-wide significant cutoff was $8.6 \times 10^{-7}$ ($0.05 / (3 \times 19{,}347)$). *p* values are determined by the upper cumulative quantile (one-sided) of Poisson distribution. Not adjusted.

**Supplementary Table 5. Top 20 significant genes in binomial test of damaging variants enrichment.**

| Gene | pLI | Observed | Expected | Fold change | p-value |
|---|---|---|---|---|---|
| *RASA1* | 1.00 | 5 | 0.18 | 28.39 | 1.20E-06 |
| *EPHB4* | 0.01 | 5 | 0.29 | 17.49 | 1.22E-05 |
| *OR51M1* | 0.00 | 2 | 0.02 | 105.87 | 1.76E-04 |
| *TAS2R14* | NA | 2 | 0.02 | 104.63 | 1.78E-04 |
| *AOC2* | 0.00 | 3 | 0.12 | 24.90 | 2.66E-04 |
| *THBS2* | 0.56 | 4 | 0.34 | 11.94 | 3.99E-04 |
| *IRF3* | 0.00 | 4 | 0.34 | 11.61 | 4.43E-04 |
| *UFC1* | 0.00 | 2 | 0.04 | 48.53 | 8.24E-04 |
| *CARD8* | 0.00 | 2 | 0.05 | 43.42 | 1.03E-03 |
| *MALSU1* | 0.00 | 2 | 0.05 | 42.88 | 1.05E-03 |
| *ZSCAN4* | 0.00 | 2 | 0.05 | 42.31 | 1.08E-03 |
| *COL27A1* | 0.59 | 4 | 0.46 | 8.72 | 1.27E-03 |
| *MRPL22* | 0.00 | 2 | 0.05 | 37.95 | 1.34E-03 |
| *C11ORF68* | NA | 2 | 0.06 | 35.32 | 1.52E-03 |
| *ABHD17A* | 0.02 | 2 | 0.06 | 32.79 | 1.78E-03 |
| *TCAP* | 0.19 | 2 | 0.06 | 31.99 | 1.87E-03 |
| *CGREF1* | 0.00 | 2 | 0.07 | 28.61 | 2.33E-03 |
| *DFNA5* | 0.00 | 2 | 0.07 | 28.52 | 2.34E-03 |
| *KLHL30* | 0.00 | 3 | 0.26 | 11.44 | 2.47E-03 |
| *KRT13* | 0.00 | 3 | 0.27 | 11.16 | 2.64E-03 |

pLI is a gene-wide constraint metric that estimates the probability of being intolerant to LoF variants based on gnomAD v2.1.1. Mutations in *RASA1* showed a genome-wide significant enrichment (one-tailed binomial test, Bonferroni multiple testing threshold = $2.6 \times 10^{-6}$) of analysis of damaging variants in all genes.

**Supplementary Table 6. Top 20 significant genes in case-control burden test of damaging variants.**

| Gene | N_alt_cases | N_ref_cases | N_alt_ctrls | N_ref_ctrls | OR | L95 | U95 | p-value |
|---|---|---|---|---|---|---|---|---|
| *RASA1* | 5 | 223 | 90 | 270680 | 67.50 | 25.76 | Inf | 2.20E-08 |
| *EPHB4* | 5 | 223 | 222 | 271182 | 27.39 | 10.60 | Inf | 1.65E-06 |
| *CAST* | 4 | 224 | 127 | 270907 | 38.11 | 12.72 | Inf | 5.24E-06 |
| *SUPT20HL2* | 2 | 226 | 5 | 176154 | 309.42 | 43.71 | Inf | 3.48E-05 |
| *ARHGAP21* | 4 | 224 | 228 | 271138 | 21.24 | 7.14 | Inf | 4.90E-05 |
| *UFC1* | 2 | 226 | 27 | 271421 | 88.95 | 14.99 | Inf | 2.80E-04 |
| *FARP1* | 5 | 223 | 706 | 270752 | 8.60 | 3.35 | Inf | 3.68E-04 |
| *ABHD17A* | 2 | 226 | 34 | 260518 | 67.71 | 11.53 | Inf | 4.70E-04 |
| *IRF3* | 4 | 224 | 468 | 270984 | 10.34 | 3.49 | Inf | 7.26E-04 |
| *SUCLA2* | 3 | 225 | 206 | 271072 | 17.54 | 4.71 | Inf | 7.72E-04 |
| *MRPL22* | 2 | 226 | 48 | 271352 | 50.06 | 8.60 | Inf | 8.37E-04 |
| *GATD3A* | 1 | 227 | 0 | 271486 | Inf | 62.68 | Inf | 8.39E-04 |
| *GOLGA6L7* | 1 | 227 | 0 | 271486 | Inf | 62.68 | Inf | 8.39E-04 |
| *GVQW3* | 1 | 227 | 0 | 271486 | Inf | 62.68 | Inf | 8.39E-04 |
| *MARCHF10* | 1 | 227 | 0 | 271486 | Inf | 62.68 | Inf | 8.39E-04 |
| *NOTCH2NLA* | 1 | 227 | 0 | 271486 | Inf | 62.68 | Inf | 8.39E-04 |
| *SDR42E2* | 1 | 227 | 0 | 271486 | Inf | 62.68 | Inf | 8.39E-04 |
| *SEPTIN1* | 1 | 227 | 0 | 271486 | Inf | 62.68 | Inf | 8.39E-04 |
| *SEPTIN10* | 1 | 227 | 0 | 271486 | Inf | 62.68 | Inf | 8.39E-04 |
| *SEPTIN9* | 1 | 227 | 0 | 271486 | Inf | 62.68 | Inf | 8.39E-04 |

Case-control burden analysis comparing all rare (minor allele frequency [MAF] ⩽ 5x10-5) protein-damaging mutations in 114 VOGM cases to (gnomAD-TOPMed) controls. Gene intolerance scores, the total number of alternative alleles, the total number of reference alleles, the alternative allele frequency in cases and controls as well as the one-tailed Fisher's exact p-value (not adjusted) comparing the allele frequencies between cases and controls are shown. The genome-wide significant cutoff is 0.05/19,347 = $2.6 \times 10^{-6}$.

**Supplementary Table 7. Phenotypes of probands with variants in *RASA1* and *EPHB4.***

| ID | Class | Gene | Type | CVL | DD | PM | PV | CHF | HY | IH | HA | SZ | CP | SL | RD |
|---|---|---|---|---|---|---|---|---|---|---|---|---|---|---|---|
| KVOGM42-1 | Proband | *RASA1* | Transmitted | + | + | + | + | + | + | + | + | + | + | + | - |
| KVOGM42-2 | Mother | *RASA1* | / | + | - | - | - | - | - | - | - | - | - | - | - |
| KVOGM48-1 | Proband | *RASA1* | Transmitted | + | + | - | + | - | - | - | + | - | - | - | - |
| KVOGM48-1 | Mother | *RASA1* | / | + | - | - | - | - | - | - | - | - | - | - | - |
| KVOGM122-1 | Proband | *RASA1* | DNM | + | - | + | + | - | - | - | - | - | - | - | - |
| KVOGM_71-1 | Proband | *RASA1* | DNM | + | - | - | - | - | - | - | - | - | - | - | - |
| KVOGM95-1 | Proband | *RASA1* | Transmitted | - | - | - | - | - | - | - | - | - | - | - | - |
| KVOGM95-2 | Mother | *RASA1* | / | + | - | - | - | - | - | - | - | - | - | - | - |
| KVOGM18-1 | Proband | *EPHB4* | Transmitted | - | + | + | - | + | + | + | - | + | + | - | + |
| KVOGM18-3 | Father | *EPHB4* | / | - | - | - | - | - | - | - | - | - | - | - | - |
| VOGM115-1 | Proband | *EPHB4* | Transmitted | - | + | + | + | - | + | + | + | + | - | - | - |
| VOGM115-3 | Father | *EPHB4* | / | + | - | - | - | - | - | - | - | - | - | - | - |
| KVOGM25-1 | Proband | *EPHB4* | Transmitted | - | + | + | - | + | + | + | - | - | - | - | - |
| KVOGM25-3 | Father | *EPHB4* | / | - | - | - | - | - | - | - | - | - | - | - | - |
| KVOGM33-1 | Proband | *EPHB4* | Transmitted | - | + | + | + | + | - | - | - | - | - | - | - |
| KVOGM33-2 | Mother | *EPHB4* | / | + | - | - | - | - | - | - | - | - | - | - | - |
| KVOGM_72-1 | Proband | *EPHB4* | Transmitted | - | - | - | - | + | - | - | - | - | - | - | - |
| KVOGM_72-3 | Father | *EPHB4* | / | - | - | - | - | - | - | - | - | - | - | - | - |

CVL: Cutaneous vascular lesions; DD: Developmental delays; PM: Progressive macrocephaly; PV: Prominent veins on the face and scalp; CHF: Congestive heart failure; HY: Hydrocephalus; IH: Intracranial hemorrhage; HA: Headaches; SZ: Seizures; CP: Cerebral palsy; SL: Skin lesions; RD: Renal/kidney disease. "+" indicates phenotype present, "-" indicates phenotype not present.

## Supplementary Table 8. Top 10 term of gene ontologies and pathway analysis.

| Term | p-value | Adjusted p-value | Odds Ratio |
|---|---|---|---|
| Go Biological Process | | | |
| positive regulation of transcription, DNA-templated (GO:0045893) | 1.83E-13 | 5.42E-10 | 3.06 |
| axonogenesis (GO:0007409) | 1.94E-11 | 2.87E-08 | 5.73 |
| eye morphogenesis (GO:0048592) | 1.39E-10 | 1.37E-07 | 37.47 |
| heart development (GO:0007507) | 5.19E-10 | 3.06E-07 | 6.48 |
| positive regulation of transcription by RNA polymerase II (GO:0045944) | 6.02E-10 | 3.06E-07 | 2.89 |
| regulation of apoptotic process (GO:0042981) | 6.21E-10 | 3.06E-07 | 3.12 |
| membrane depolarization (GO:0051899) | 9.19E-10 | 3.88E-07 | 20.85 |
| membrane depolarization during action potential (GO:0086010) | 2.30E-09 | 8.38E-07 | 24.24 |
| positive regulation of nucleic acid-templated transcription (GO:1903508) | 2.55E-09 | 8.38E-07 | 3.50 |
| membrane depolarization during SA node cell action potential (GO:0086046) | 4.81E-09 | 1.42E-06 | 97820.00 |
| Go Molecular Function | | | |
| GTPase activator activity (GO:0005096) | 3.35E-10 | 1.52E-07 | 4.47 |
| voltage-gated cation channel activity (GO:0022843) | 2.84E-09 | 6.44E-07 | 8.47 |
| voltage-gated calcium channel activity (GO:0005245) | 4.50E-09 | 6.80E-07 | 16.99 |
| platelet-derived growth factor binding (GO:0048407) | 4.37E-08 | 4.95E-06 | 54.58 |
| protein kinase binding (GO:0019901) | 2.61E-07 | 2.37E-05 | 3.08 |
| transcription regulatory region nucleic acid binding (GO:0001067) | 1.01E-06 | 7.08E-05 | 4.30 |
| voltage-gated calcium channel activity involved in cardiac muscle cell action potential (GO:0086007) | 1.09E-06 | 7.08E-05 | 181.14 |
| calcium channel activity (GO:0005262) | 2.07E-06 | 1.17E-04 | 6.91 |
| cadherin binding (GO:0045296) | 2.58E-06 | 1.30E-04 | 3.41 |
| GTPase regulator activity (GO:0030695) | 3.88E-06 | 1.76E-04 | 3.88 |
| Go Cellular Component | | | |
| neuron projection (GO:0043005) | 5.45E-10 | 1.35E-07 | 3.51 |
| polymeric cytoskeletal fiber (GO:0099513) | 5.11E-08 | 6.31E-06 | 4.39 |
| intracellular membrane-bounded organelle (GO:0043231) | 1.09E-06 | 9.01E-05 | 1.64 |
| focal adhesion (GO:0005925) | 1.49E-06 | 9.20E-05 | 3.23 |
| cell-substrate junction (GO:0030055) | 2.06E-06 | 1.02E-04 | 3.16 |
| nucleus (GO:0005634) | 7.27E-06 | 2.99E-04 | 1.60 |
| cytoskeleton (GO:0005856) | 9.07E-06 | 3.20E-04 | 2.56 |
| axon (GO:0030424) | 1.10E-05 | 3.40E-04 | 3.93 |
| basement membrane (GO:0005604) | 1.55E-05 | 4.26E-04 | 8.29 |
| voltage-gated sodium channel complex (GO:0001518) | 2.40E-05 | 5.92E-04 | 18.90 |
| Wiki Pathway | | | |
| Fragile X Syndrome WP4549 | 8.72E-09 | 3.39E-06 | 7.06 |
| Focal Adhesion WP306 | 3.68E-07 | 4.92E-05 | 4.64 |
| Disruption of postsynaptic signaling by CNV WP4875 | 4.12E-07 | 4.92E-05 | 14.61 |
| MAPK Signaling Pathway WP382 | 5.06E-07 | 4.92E-05 | 4.11 |
| Notch Signaling Pathway Netpath WP61 | 6.51E-06 | 4.67E-04 | 7.91 |
| Signaling of Hepatocyte Growth Factor Receptor WP313 | 7.21E-06 | 4.67E-04 | 11.81 |
| Rett syndrome causing genes WP4312 | 8.40E-06 | 4.67E-04 | 9.12 |
| Pathways affected in adenoid cystic carcinoma WP3651 | 1.11E-05 | 5.40E-04 | 7.34 |
| Canonical and non-canonical Notch signaling WP3845 | 2.08E-05 | 8.99E-04 | 12.99 |
| NOTCH1 regulation of endothelial cell calcification WP3413 | 2.40E-05 | 9.14E-04 | 18.90 |
| Reactome Pathway | | | |
| Developmental Biology Homo sapiens R-HSA-1266738 | 1.34E-15 | 8.82E-13 | 3.89 |
| Axon guidance Homo sapiens R-HSA-422475 | 2.17E-15 | 8.82E-13 | 4.68 |
| Interaction between L1 and Ankyrins Homo sapiens R-HSA-445095 | 3.02E-10 | 8.19E-08 | 24.15 |
| L1CAM interactions Homo sapiens R-HSA-373760 | 2.45E-09 | 4.98E-07 | 8.57 |
| Signaling by PDGF Homo sapiens R-HSA-186797 | 1.33E-07 | 2.16E-05 | 3.61 |
| Extracellular matrix organization Homo sapiens R-HSA-1474244 | 2.96E-07 | 4.01E-05 | 3.93 |
| Signalling by NGF Homo sapiens R-HSA-166520 | 7.38E-07 | 8.56E-05 | 3.11 |
| Collagen biosynthesis and modifying enzymes Homo sapiens R-HSA-1650814 | 9.96E-07 | 1.01E-04 | 8.64 |
| Diseases of signal transduction Homo sapiens R-HSA-5663202 | 1.56E-06 | 1.40E-04 | 3.66 |
| NCAM signaling for neurite out-growth Homo sapiens R-HSA-375165 | 1.72E-06 | 1.40E-04 | 3.78 |

The p-values were calculated by one-sided Fisher's exact test, Bonferroni multiple-testing adjusted.

**Supplementary Table 9. Damaging variants in axon guidance genes.**

| REAC:R-hsa-422475 | D-Mis | LoF | DNV | GO:0007411 | D-Mis | LoF | DNV | Combined | D-Mis | LoF | DNV |
|---|---|---|---|---|---|---|---|---|---|---|---|
| *ALCAM* | 1 | 0 | 0 | *Agrn* | 1 | 0 | 0 | *ALCAM* | 1 | 0 | 0 |
| *ANK1* | 1 | 0 | 0 | *Alcam* | 1 | 0 | 0 | *ANK1* | 1 | 0 | 0 |
| *DAG1* | 1 | 0 | 0 | *Ank3* | 1 | 0 | 0 | *DAG1* | 1 | 0 | 0 |
| *ERBB2* | 0 | 1 | 0 | *B4gat1* | 1 | 0 | 0 | *ERBB2* | 0 | 1 | 0 |
| *ITGA1* | 0 | 2 | 0 | *Cdh4* | 1 | 1 | 0 | *ITGA1* | 0 | 2 | 0 |
| *ITGB1* | 0 | 2 | 0 | *Chl1* | 1 | 0 | 0 | *ITGB1* | 0 | 2 | 0 |
| *LAMB1* | 1 | 0 | 0 | *Cntn6* | 1 | 0 | 0 | *LAMB1* | 1 | 0 | 0 |
| *MET* | 1 | 0 | 0 | *Crppa* | 1 | 0 | 0 | *MET* | 1 | 0 | 0 |
| *MYO9B* | 1 | 0 | 0 | *Cyfip1* | 0 | 1 | 0 | *MYO9B* | 1 | 0 | 0 |
| *MYO10* | 2 | 1 | 0 | *Dag1* | 1 | 0 | 0 | *MYO10* | 2 | 1 | 0 |
| *NRCAM* | 1 | 0 | 0 | *Dscaml1* | 1 | 0 | 0 | *NRCAM* | 1 | 0 | 0 |
| *PIK3CD* | 2 | 0 | 0 | *Efnb2* | 1 | 0 | 0 | *PIK3CD* | 2 | 0 | 0 |
| *PLCG1* | 0 | 1 | 0 | *Epha4* | 1 | 0 | 0 | *PLCG1* | 0 | 1 | 0 |
| *PLXNB1* | 0 | 2 | 1 | *Epha6* | 1 | 0 | 0 | *PLXNB1* | 0 | 2 | 1 |
| *PRKAR2A* | 1 | 0 | 0 | *Epha8* | 0 | 1 | 0 | *PRKAR2A* | 1 | 0 | 0 |
| *PRNP* | 1 | 0 | 0 | *Epha10* | 1 | 0 | 0 | *PRNP* | 1 | 0 | 0 |
| *RELN* | 1 | 0 | 0 | *Ephb4* | 4 | 1 | 0 | *RELN* | 1 | 0 | 0 |
| *PSMC3* | 1 | 0 | 0 | *Erbb2* | 0 | 1 | 0 | *PSMC3* | 1 | 0 | 0 |
| *PSMD13* | 1 | 0 | 0 | *Evl* | 1 | 0 | 0 | *PSMD13* | 1 | 0 | 0 |
| *PTK2* | 2 | 0 | 0 | *Ext1* | 1 | 0 | 0 | *PTK2* | 2 | 0 | 0 |
| *PTPN11* | 1 | 0 | 1 | *Gbx2* | 1 | 0 | 0 | *PTPN11* | 1 | 0 | 1 |
| *RASA1* | 0 | 4 | 2 | *Lama1* | 1 | 0 | 0 | *RASA1* | 0 | 4 | 2 |
| *RPL12* | 0 | 1 | 0 | *Lama2* | 1 | 0 | 0 | *RPL12* | 0 | 1 | 0 |
| *ITSN1* | 1 | 0 | 0 | *Lama3* | 3 | 0 | 0 | *ITSN1* | 1 | 0 | 0 |
| *SLIT3* | 1 | 0 | 0 | *Lama5* | 2 | 1 | 0 | *SLIT3* | 1 | 0 | 0 |
| *VAV2* | 1 | 0 | 0 | *Lgr4* | 0 | 1 | 0 | *VAV2* | 1 | 0 | 0 |
| *SEMA7A* | 1 | 0 | 0 | *Lrp1* | 2 | 0 | 0 | *SEMA7A* | 1 | 0 | 0 |
| *UNC5C* | 1 | 0 | 0 | *Lrp2* | 3 | 0 | 0 | *UNC5C* | 1 | 0 | 0 |
| *NRP2* | 0 | 9 | 0 | *Nexn* | 0 | 1 | 0 | *NRP2* | 0 | 9 | 0 |
| *NRP1* | 0 | 1 | 0 | *Nfasc* | 1 | 0 | 0 | *NRP1* | 0 | 1 | 0 |
| *SLIT2* | 1 | 0 | 0 | *Notch1* | 3 | 0 | 1 | *SLIT2* | 1 | 0 | 0 |
| *FARP2* | 3 | 0 | 0 | *Notch3* | 4 | 0 | 0 | *FARP2* | 3 | 0 | 0 |
| *CHL1* | 1 | 0 | 0 | *Nrcam* | 1 | 0 | 0 | *CHL1* | 1 | 0 | 0 |
| *NFASC* | 1 | 0 | 0 | *Nrp1* | 0 | 1 | 0 | *NFASC* | 1 | 0 | 0 |
| *NGEF* | 1 | 0 | 0 | *Nrp2* | 0 | 9 | 0 | *NGEF* | 1 | 0 | 0 |
| *CNTN6* | 1 | 0 | 0 | *Or10a4* | 0 | 1 | 0 | *CNTN6* | 1 | 0 | 0 |
| *GFRA4* | 0 | 1 | 0 | *Plxnb1* | 0 | 2 | 1 | *GFRA4* | 0 | 1 | 0 |
| *AGAP2* | 1 | 0 | 0 | *Reln* | 1 | 0 | 0 | *AGAP2* | 1 | 0 | 0 |
| *PSMB11* | 0 | 1 | 0 | *Sema3g* | 0 | 1 | 0 | *PSMB11* | 0 | 1 | 0 |
| *LAMA1* | 1 | 0 | 0 | *Sema7a* | 1 | 0 | 0 | *LAMA1* | 1 | 0 | 0 |
| *AGRN* | 1 | 0 | 0 | *Shh* | 1 | 0 | 0 | *AGRN* | 1 | 0 | 0 |
| *CD24* | 0 | 11 | 0 | *Slit2* | 1 | 0 | 0 | *CD24* | 0 | 11 | 0 |
| | | | | *Slit3* | 1 | 0 | 0 | *Ank3* | 1 | 0 | 0 |
| | | | | *Smo* | 1 | 0 | 0 | *B4gat1* | 1 | 0 | 0 |
| | | | | *Tenm2* | 1 | 0 | 0 | *Cdh4* | 1 | 1 | 0 |
| | | | | *Unc5c* | 1 | 0 | 0 | *Crppa* | 1 | 0 | 0 |
| | | | | | | | | *Cyfip1* | 0 | 1 | 0 |
| | | | | | | | | *Dscaml1* | 1 | 0 | 0 |
| | | | | | | | | *Efnb2* | 1 | 0 | 0 |
| | | | | | | | | *Epha4* | 1 | 0 | 0 |
| | | | | | | | | *Epha6* | 1 | 0 | 0 |
| | | | | | | | | *Epha8* | 0 | 1 | 0 |
| | | | | | | | | *Epha10* | 1 | 0 | 0 |
| | | | | | | | | *Ephb4* | 4 | 1 | 0 |
| | | | | | | | | *Evl* | 1 | 0 | 0 |
| | | | | | | | | *Ext1* | 1 | 0 | 0 |
| | | | | | | | | *Gbx2* | 1 | 0 | 0 |
| | | | | | | | | *Lama2* | 1 | 0 | 0 |
| | | | | | | | | *Lama3* | 3 | 0 | 0 |
| | | | | | | | | *Lama5* | 2 | 1 | 0 |
| | | | | | | | | *Lgr4* | 0 | 1 | 0 |
| | | | | | | | | *Lrp1* | 2 | 0 | 0 |
| | | | | | | | | *Lrp2* | 3 | 0 | 0 |
| | | | | | | | | *Nexn* | 0 | 1 | 0 |
| | | | | | | | | *Notch1* | 3 | 0 | 1 |
| | | | | | | | | *Notch3* | 4 | 0 | 0 |
| | | | | | | | | *Or10a4* | 0 | 1 | 0 |
| | | | | | | | | *Sema3g* | 0 | 1 | 0 |
| | | | | | | | | *Shh* | 1 | 0 | 0 |
| | | | | | | | | *Smo* | 1 | 0 | 0 |
| | | | | | | | | *Tenm2* | 1 | 0 | 0 |
| Total | 35 | 37 | 4 | Total | 50 | 22 | 2 | Total | 72 | 46 | 5 |

D-Mis: number of damaging missense mutations as predicted by MetaSVM or MPC-2; LoF: number of loss-of-function mutations comprised of premature termination, frameshift or splice site mutation; DNV: number of damaging de novo variants.

**Supplementary Table 10. Characteristics of patients with *de novo* and transmitted variant in other interesting genes.**

| Proband ID | Sex | Ethnicity | Position (GRCH37) | Proband GT | Father GT | Mother GT | Gene | Class | AA Change | Type |
|---|---|---|---|---|---|---|---|---|---|---|
| VOGM100-1* | Female | Mexican | 12:52314616:G:A | 0/1 | 0/1 | 0/0 | *ACVRL1* | Transmitted | p.R484Q | D-Mis |
| KVOGM91-1 | Female | European | 12:52309267:G:A | 0/1 | 0/1 | 0/0 | *ACVRL1* | Transmitted | p.C344Y | D-Mis |
| VOGM105-1* | Male | European | 10:33196073:T:C | 0/1 | 0/1 | 0/1 | *ITGB1* | Transmitted | c.2331-2 A>G | LoF |
| KVOGM89-1 | Male | European | 10:33196039: ATTAATAGGAC:A | 0/1 | NA | NA | *ITGB1* | Unphased | p.S785fs | LoF |
| KVOGM_73-1 | Female | European | 9:139409992:C:A | 0/1 | 0/0 | 0/0 | *NOTCH1* | DNV | p.G616C | D-Mis |
| VOGM46-1 | Male | French | 9:139391554:C:T | 0/1 | 0/0 | 0/1 | *NOTCH1* | Transmitted | p.V2213M | D-Mis |
| KVOGM83-1 | Male | European | 9:139402819:C:T | 0/1 | NA | NA | *NOTCH1* | Unphased | p.D1064N | D-Mis |
| KVOGM23-1 | Female | European | 12:112888172:A:G | 0/1 | 0/0 | 0/0 | *PTPN11* | DNV | p.Y63C | D-Mis |

*Indicate patients have been reported in our previous study[1]; NA: not applicable; GT: genotype; DNV: *de novo* variants; D-mis: missense mutations as predicted by MetaSVM or MPC-2; LoF: loss-of-function mutations comprised of premature termination, frameshift or splice site mutation.

**Supplementary Table 11. Summary of clinical features of patients with *de novo* and transmitted variant in other interesting genes.**

| Proband ID | Gene | DD | HY | CHF | PM | IH | PV | SZ | HA | RN |
|---|---|---|---|---|---|---|---|---|---|---|
| VOGM100-1* | *ACVRL1* | + | + | + | - | - | - | + | - | + |
| KVOGM91-1 | *ACVRL1* | + | + | + | - | - | - | - | - | - |
| VOGM105-1* | *ITGB1* | - | + | - | + | + | - | + | + | - |
| KVOGM89-1 | *ITGB1* | + | - | - | + | + | + | - | - | - |
| KVOGM_73-1 | *NOTCH1* | + | - | + | - | + | - | - | - | - |
| VOGM46-1 | *NOTCH1* | - | - | - | + | - | + | - | - | - |
| KVOGM83-1 | *NOTCH1* | - | - | - | - | - | - | - | - | - |
| KVOGM23-1 | *PTPN11* | - | + | - | - | - | - | - | - | - |

*Indicate patients have been reported in our previous study[1]; DD: Developmental delays; HY: Hydrocephalus; CHF: Congestive heart failure; PM: Progressive macrocephaly; IH: Intracranial hemorrhage; PV: Prominent veins on the face and scalp; SZ: Seizures; HA: Headaches; RN: Recurrent nosebleeds. "+" indicates phenotype present, "-" indicates phenotype not present.

**Supplementary Table 12. Damaging variants in Ras signaling pathway.**

| WP4223 (Ras signaling) | D-Mis | LoF | DNV |
|---|---|---|---|
| *AKT1* | 5 | 11 | 0 |
| *AKT2* | 1 | 0 | 0 |
| *FGFR2* | 1 | 0 | 0 |
| *FGFR4* | 1 | 0 | 0 |
| *GNG11* | 0 | 1 | 0 |
| *INSR* | 1 | 0 | 0 |
| *KIT* | 1 | 0 | 0 |
| *MET* | 1 | 0 | 0 |
| *NF1* | 1 | 0 | 0 |
| *PIK3CD* | 2 | 0 | 0 |
| *PLCG1* | 0 | 1 | 0 |
| *PLCG2* | 1 | 0 | 0 |
| *PTPN11* | 1 | 0 | 1 |
| *RAP1A* | 1 | 0 | 0 |
| *RASA1* | 0 | 4 | 2 |
| *RASGRF1* | 1 | 0 | 0 |
| *SHOC2* | 1 | 0 | 0 |
| *RASAL1* | 1 | 1 | 0 |
| *SYNGAP1* | 1 | 0 | 0 |
| *KSR1* | 9 | 0 | 0 |
| *RASAL2* | 1 | 0 | 0 |
| *PAK4* | 1 | 0 | 0 |
| *PLA2G12A* | 1 | 0 | 0 |
| Total | 33 | 18 | 3 |

D-Mis: number of damaging missense mutations as predicted by MetaSVM or MPC-2; LoF: number of loss-of-function mutations comprised of premature termination, frameshift or splice site mutation; DNV: number of damaging de novo variants.

**Supplementary Table 13. Module gene sets.**

| Gene Set | Gene | Disorder | Reference |
|---|---|---|---|
| AVM | *KRAS* | Arteriovenous malformation of the brain, somatic (OMIM:108010) | PMID: 30382944 |
| | *NRAS* | Noonan syndrome (OMIM:6 613224) | PMID: 30382944 |
| | *BRAF* | Noonan syndrome (OMIM:7 613706) | PMID: 30382944 |
| | *MAP2K1* | Cardiofaciocutaneous syndrome (OMIM:3 615279) | PMID: 30382944 |
| | *NOTCH4* | NA | PMID: 24373503 |
| | *EPHB4* | Capillary malformation-arteriovenous malformation 2 (OMIM:618196) | PMID: 30819650 |
| | *RASA1* | Capillary malformation-arteriovenous malformation 1 (OMIM:608354) | PMID: 30819650 |
| | *ALK1 (ACVRL1)* | Telangiectasia, hereditary hemorrhagic, type 2 (OMIM:600376) | PMID: 31731545 |
| | *SMAD4* | Juvenile polyposis/hereditary hemorrhagic telangiectasia syndrome (OMIM:175050) | PMID: 31731545 |
| | *ENG* | Telangiectasia, hereditary hemorrhagic, type (OMIM: 1 187300) | PMID: 31731545 |
| | *GNAQ* | Sturge-Weber syndrome, somatic, mosaic (OMIM: 185300) | PMID: 28126187 |
| CCM | *CCM1/KRIT1* | Cerebral cavernous malformations 1 (OMIM:116860) | PMID: 19574835 |
| | *CCM2/MGC4607* | Cerebral cavernous malformations 2 (OMIM:603284) | PMID: 19574835 |
| | *CCM3/PDCD10* | Cerebral cavernous malformations 3 (OMIM:603285) | PMID: 19574835 |
| | *PIK3CA* | Cerebral cavernous malformations 4, somatic (OMIM:619538) | PMID: 34496175 |
| | *MAP3K3* | NA | PMID: 33891857 |
| VOGM | *RASA1* | Capillary malformation-arteriovenous malformation 1 (OMIM:608354) | PMID: 29350590 |
| | *EPHB4* | Capillary malformation-arteriovenous malformation 2 (OMIM:618196) | PMID: 30578106 |
| | *ENG* | Telangiectasia, hereditary hemorrhagic, type 1 (OMIM:187300) | PMID: 29350590 |
| | *ACVRL1* | Telangiectasia, hereditary hemorrhagic, type 2 (OMIM:600376) | PMID: 32170914 |
| pVOGM | *NOTCH1* | Aortic valve disease 1 (OMIM:109730) | NA |
| | *RASA1* | Capillary malformation-arteriovenous malformation 1 (OMIM:608354) | NA |
| | *PTPN11* | Noonan syndrome 1 (OMIM:163950) | NA |
| | *MUC5B* | NA | NA |
| | *KMT2D* | Kabuki syndrome 1 (OMIM:147920) | NA |
| | *RAB11FIP4* | NA | NA |
| | *SMARCA1* | NA | NA |
| | *KAT6A* | Arboleda-Tham syndrome (OMIM:616268) | NA |
| | *ANK2* | Cardiac arrhythmia, ankyrin-B-related (OMIM:600919) | NA |
| | *LPGAT1* | NA | NA |
| | *EPHB4* | Capillary malformation-arteriovenous malformation 2 (OMIM:618196) | NA |
| | *ENG* | Telangiectasia, hereditary hemorrhagic, type 1 (OMIM:187300) | NA |
| | *ACVRL1* | Telangiectasia, hereditary hemorrhagic, type 2 (OMIM:600376) | NA |
| MMD | *RNF213* | Moyamoya disease 2 (OMIM:607151) | PMID: 23466837 |
| | *GUCY1A3* | Moyamoya 6 with achalasia (OMIM:615750) | PMID: 26777256 |
| | *BRCC3* | NA | PMID: 21596366 |
| | *CMC4/MTPC1* | NA | PMID: 21596366 |
| | *DIAPH1* | NA | PMID: 34125151 |
| | *CNOT3* | Intellectual developmental disorder with speech delay, autism, and dysmorphic facies (OMIM:618672) | PMID: 31474762 |
| | *ACTA2* | Moyamoya disease 5 (OMIM:614042) | PMID: 20970362 |

NA: not applicable; AVM: arteriovenous malformation; CCM: cavernous malformation; VOGM: vein of Galen aneurysmal malformation; MMD: moyamoya disease; pVOGM: possible VOGM genes (VOGM gene set + DNVs with pLI⩾0.9); height gene set referenced from PMID: 20881960[4].

**Supplementary Table 14. Damaging recessive mutations in 114 VOGM cases.**

| Proband ID | Proband GT | Father GT | Mother GT | Mutation Type | Variant (GRCH37) | Gene | Type | AA Change | OMIM Inheritance |
|---|---|---|---|---|---|---|---|---|---|
| KVOGM74-1 | 0/1 | 0/1 | 0/0 | CompHet | 4:983279:A:T | *SLC26A1* | D-mis | p.L483Q | AR |
| KVOGM74-1 | 0/1 | 0/0 | 0/1 | CompHet | 4:983432:G:A | *SLC26A1* | D-mis | p.P432L | AR |
| VOGM111-1 | 0/1 | 0/0 | 0/1 | CompHet | 19:49387033:C:T | *TULP2* | D-mis | p.R418Q | N/A |
| VOGM111-1 | 0/1 | 0/1 | 0/0 | CompHet | 19:49388791:T:G | *TULP2* | D-mis | p.R325S | N/A |
| KVOGM34-1 | 0/1 | 0/1 | 0/0 | CompHet | 2:179419389:C:T | *TTN* | D-mis | p.G20689D | AD or AR |
| KVOGM34-1 | 0/1 | 0/0 | 0/1 | CompHet | 2:179449579:C:T | *TTN* | D-mis | p.V12724M | AD or AR |
| KVOGM53-1 | 0/1 | 0/0 | 0/1 | CompHet | 2:179391815:C:T | *TTN* | D-mis | p.G27094E | AD or AR |
| KVOGM53-1 | 0/1 | 0/1 | 0/0 | CompHet | 2:179434691:G:T | *TTN* | D-mis | p.P16517T | AD or AR |
| VOGM100-1 | 0/1 | 0/0 | 0/1 | CompHet | 11:93754578:T:C | *HEPHL1* | D-mis | p.F15S | AR |
| VOGM100-1 | 0/1 | 0/1 | 0/0 | CompHet | 11:93800682:G:A | *HEPHL1* | D-mis | p.G277R | AR |
| KVOGM12-1 | 0/1 | 0/1 | 0/0 | CompHet | 1:160125881:G:A | *ATP1A4* | D-mis | p.C153Y | N/A |
| KVOGM12-1 | 0/1 | 0/0 | 0/1 | CompHet | 1:160136430:G:A | *ATP1A4* | D-mis | p.G387D | N/A |
| KVOGM4-1 | 0/1 | 0/1 | 0/0 | CompHet | 7:48567844:C:G | *ABCA13* | D-mis | p.L4753V | N/A |
| KVOGM4-1 | 0/1 | 0/0 | 0/1 | CompHet | 7:48567855:T:A | *ABCA13* | D-mis | p.N4756K | N/A |
| KVOGM44-1 | 0/1 | 0/1 | 0/0 | CompHet | 2:141739813:C:T | *LRP1B* | D-mis | p.G935R | N/A |
| KVOGM44-1 | 0/1 | 0/0 | 0/1 | CompHet | 2:141946030:G:A | *LRP1B* | D-mis | p.L325F | N/A |
| VOGM103-1 | 1/1 | NA | 0/1 | Homozygous | 1:32138081:G:A | *COL16A1* | D_mis | p.P1014L | N/A |
| KVOGM_73-1 | 1/1 | 0/1 | 0/1 | Homozygous | 2:38527451:T:C | *ATL2* | D_mis | p.Q364R | N/A |
| KVOGM1-1 | 1/1 | 0/0 | 0/0 | Homozygous | 2:42275671:G:T | *PKDCC* | D_mis | p.G111V | AR |
| KVOGM52-1 | 1/1 | 0/1 | 0/1 | Homozygous | 2:206641245:T:TA | *NRP2* | stopgain | p.C901* | N/A |
| KVOGM_73-1 | 1/1 | 0/1 | 0/1 | Homozygous | 9:98242721:G:A | *PTCH1* | D_mis | p.P148L | AD |
| KVOGM_73-1 | 1/1 | 0/1 | 1/1 | Homozygous | 10:69991251:G:T | *ATOH7* | D_mis | p.R62S | AR |
| KVOGM_57-1 | 1/1 | NA | 0/1 | Homozygous | 14:59798063:C:T | *DAAM1* | D_mis | p.P566L | N/A |
| KVOGM88-1 | 1/1 | NA | NA | Homozygous | 15:84639249:G:A | *ADAMTSL3* | D_mis | p.C835Y | N/A |
| KVOGM_72-1 | 1/1 | 0/1 | NA | Homozygous | 17:40312063:G:A | *KCNH4* | D_mis | p.H1017Y | N/A |
| KVOGM88-1 | 1/1 | NA | NA | Homozygous | 19:2878195:CA:C | *ZNF556* | frameshift | p.N415Mfs*2 | N/A |
| KVOGM_57-1 | 1/1 | NA | 0/1 | Homozygous | 20:42252537:G:C | *IFT52* | D_mis | p.D83H | AR |
| KVOGM_57-1 | 1/1 | NA | 0/1 | Homozygous | 22:21134442:T:C | *SERPIND1* | D_mis | p.I281T | AD |

GT: genotype; NA: not applicable; CompHet: compound heterozygous variants; D-mis: missense mutations as predicted by MetaSVM or MPC-2; AR: Autosomal recessive; AD: Autosomal dominant.

**Supplementary Table 15. Gene-specific crRNA sequences designed using CRISPR.**

| Gene | crRNA | Forward primer | Reverse primer |
|---|---|---|---|
| *ephb4a* | AGAATCCACGCTTGCTCAAC | TAATTGTGTTTTTATTCTCCAGGTG | TATTTCCAATCTTAAATTAGCTTGC |
| *ephb4a* | CAGGATCCTGTAGTGTGACT | CCATGCGGCTAGTGAAGGAGC | CTTCTCGCAGTAGCGTATCTGG |
| *ephb4b* | AGCGTCGCAGTATCAGGTGC | GTAATAGTGTTGTCACTATACTGTC | TAAGTTAGTGTGCATGATATGTATG |
| *ephb4b* | GCCATCAAGACGCTGAAGGG | CATGTCCATCTGATATTCCTCATAC | AGAGAAATAAACAGATAGACGAATG |
| *acvrl1* | CACCTGGATGGGAGAAAGTG | CAGTGTGGGTGGAACTTAC | GCTCTGATTGCTTCTGGTAG |
| *acvrl1* | CCAGGTCTGCAATGCAGCAC | ATAATATGTTTTGACATTGCCAAAC | CCTAAATAAACCAAACAATATGAGC |
| *acvrl1 scramble* | AGAGGTCGGAAGCTAGGCAT | CAGTGTGGGTGGAACTTAC | GCTCTGATTGCTTCTGGTAG |
| *acvrl1 scramble* | GATAACGCCTTCGCGCAGCA | ATAATATGTTTTGACATTGCCAAAC | CCTAAATAAACCAAACAATATGAGC |

## References:


1 Duran, D. *et al.* Mutations in Chromatin Modifier and Ephrin Signaling Genes in Vein of Galen Malformation. *Neuron* **101**, 429-443.e424, doi:10.1016/j.neuron.2018.11.041 (2019).

2 Tröster, A. *et al.* NVP-BHG712: Effects of Regioisomers on the Affinity and Selectivity toward the EPHrin Family. *ChemMedChem* **13**, 1629-1633, doi:10.1002/cmdc.201800398 (2018).

3 Wybenga-Groot, L. E. *et al.* Structural basis for autoinhibition of the Ephb2 receptor tyrosine kinase by the unphosphorylated juxtamembrane region. *Cell* **106**, 745-757, doi:10.1016/s0092-8674(01)00496-2 (2001).

4 Lango Allen, H. *et al.* Hundreds of variants clustered in genomic loci and biological pathways affect human height. *Nature* **467**, 832-838, doi:10.1038/nature09410 (2010).
