# Supplementary material for: Mutation of key signaling regulators of cerebrovascular development in vein of Galen malformations
Source: Nat Commun. 2023 Nov 17;14:7452. doi: 10.1038/s41467-023-43062-z (PMC10656524; doi:10.1038/s41467-023-43062-z)
Supplement: Supplementary file 3 — Description of Additional Supplementary Files [file 41467_2023_43062_MOESM3_ESM.docx]

**Description of Additional Supplementary Files**

File Name: Supplementary Data 1

Description: Proband information for 114 VOGM cases

File Name: Supplementary Data 2

Description: *De novo* variants in 90 VOGM parents-proband trios

File Name: Supplementary Data 3

Description: Distribution of highly pathogenic mutations in 114 VOGM patients with different sub-phenotypes
