# Supplementary material for: Mutation of key signaling regulators of cerebrovascular development in vein of Galen malformations
Source: Nat Commun. 2023 Nov 17;14:7452. doi: 10.1038/s41467-023-43062-z (PMC10656524; doi:10.1038/s41467-023-43062-z)
Supplement: Supplementary file 7 — Reporting Summary [file 41467_2023_43062_MOESM7_ESM.pdf]

nature portfolio

Corresponding author(s): Kristopher T. Kahle, MD PhD

Last updated by author(s): Oct 10, 2023

Reporting Summary

Nature Portfolio wishes to improve the reproducibility of the work that we publish. This form provides structure for consistency and transparency in reporting. For further information on Nature Portfolio policies, see our Editorial Policies and the Editorial Policy Checklist.

Statistics

For all statistical analyses, confirm that the following items are present in the figure legend, table legend, main text, or Methods section.

n/a	Confirmed	
☐	☒	The exact sample size (*n*) for each experimental group/condition, given as a discrete number and unit of measurement
☐	☒	A statement on whether measurements were taken from distinct samples or whether the same sample was measured repeatedly
☐	☒	The statistical test(s) used AND whether they are one- or two-sided<br>*Only common tests should be described solely by name; describe more complex techniques in the Methods section.*
☐	☒	A description of all covariates tested
☐	☒	A description of any assumptions or corrections, such as tests of normality and adjustment for multiple comparisons
☐	☒	A full description of the statistical parameters including central tendency (e.g. means) or other basic estimates (e.g. regression coefficient) AND variation (e.g. standard deviation) or associated estimates of uncertainty (e.g. confidence intervals)
☐	☒	For null hypothesis testing, the test statistic (e.g. *F*, *t*, *r*) with confidence intervals, effect sizes, degrees of freedom and *P* value noted<br>*Give P values as exact values whenever suitable.*
☐	☒	For Bayesian analysis, information on the choice of priors and Markov chain Monte Carlo settings
☒	☐	For hierarchical and complex designs, identification of the appropriate level for tests and full reporting of outcomes
☒	☐	Estimates of effect sizes (e.g. Cohen's *d*, Pearson's *r*), indicating how they were calculated

Our web collection on statistics for biologists contains articles on many of the points above.

Software and code

Policy information about availability of computer code

Data collection	HiSeq (Illumina) 2000, 2500, 4000 or NovaSeq 6000
Data analysis	Software utilized in this study is available at the following web addresses: Samtools v1.3.1 (https://github.com/samtools/samtools); GATK HaplotypeCaller v3.7.0 (https://github.com/broadinstitute/gatk/releases); GATK GenotypeGVCFs v3.7.0 (https://github.com/broadinstitute/gatk/releases); GATK VariantRecalibrator v3.7.0 (https://github.com/broadinstitute/gatk/releases); TrioDeNovo v0.6.0 (http://genome.sph.umich.edu/wiki/Triodenovo); DenovolyzeR v0.2.0 (http://denovolyzer.org); Plink v1.9 (http://pngu.mgh.harvard.edu/~purcell/plink); MetaSVM/CADD13/ANNOVAR v4.2 (http://annovar.openbioinformatics.org); R v3.5.0 (https://www.r-project.org/); Python v2.7 (https://www.python.org/downloads/); EIGENSTRAT v7.2.1 (https://github.com/DReichLab/EIG/tree/master/EIGENSTRAT); EnrichR R package v3.0 (https://cran.r-project.org/web/packages/enrichR/index.html); Monocle R package Version 3 (https://cole-trapnell-lab.github.io/monocle3/); Our in-house pipelines and codes are available at https://github.com/Kahle-Lab/VOGM.

For manuscripts utilizing custom algorithms or software that are central to the research but not yet described in published literature, software must be made available to editors and reviewers. We strongly encourage code deposition in a community repository (e.g. GitHub). See the Nature Portfolio guidelines for submitting code & software for further information.

Data

Policy information about availability of data

All manuscripts must include a data availability statement. This statement should provide the following information, where applicable:

- Accession codes, unique identifiers, or web links for publicly available datasets
- A description of any restrictions on data availability
- For clinical datasets or third party data, please ensure that the statement adheres to our policy

The raw sequencing data for all VOGM samples in fastq format generated in this study have been deposited in the NCBI database of Genotypes and Phenotypes as well as AnVIL under accession code phs000744.v5.p2 (https://www.ncbi.nlm.nih.gov/projects/gap/cgi-bin/study.cgi?study_id=phs000744.v5.p2). Genomic and phenotypic data for 1,798 unaffected siblings of autism cases and unaffected parents from the SSC can be accessed through SFARI Base (https://base.sfari.org/) with an approved application. Reference genome used in this study is genome assembly GRCh37/hg19 (https://www.ncbi.nlm.nih.gov/datasets/genome/GCF_000001405.13/). Additionally, the data from the mice used in this study can be found in the Supplementary Information/Source Data file. Source data for this paper are provided herein.

Human research participants

Policy information about studies involving human research participants and Sex and Gender in Research.

Reporting on sex and gender	Biological sex was determined by EIGENSTRAT. Male sex/female sex percentages: 65.8%/34.2%. Self-reported sex are listed in Supplementary Data 1.
Population characteristics	114 probands diagnosed with VOGM were included in this study. Age of diagnosis was as follows: Prenatal: 34.21%; Postnatal Neonate: 20.18%; Infant: 28.07%; Young child: 2.63%; Child: 0.88%; Unknown: 47.37%. Self reported race was as follows: African: 5.26%; Asian: 7.02%; Caucasian: 64.04%; Hispanic: 0.88%; Mixed Race: 8.77%. Associated conditions was as follows: High output heart failure: 40.35%; Progressive macrocephaly: 48.25%; Developmental delay: 54.39%; Hydrocephalus: 48.25%; Headaches: 21.93%; Intracranial hemorrhage: 21.05%; Prominent veins on face and scalp: 44.74%; Recurrent nosebleeds: 4.39%; Renal/kidney disease: 2.63%; Seizures: 31.58%. 36.0% had a family history of cutaneous vascular abnormalities. See Supplementary Data 1 and Supplementary Table 1 for details.
Recruitment	Patients were recruited from multiple academic health care institutions and social media. Inclusion criteria included male or female patients with a clearly defined mural or choroidal VOGMs, radiographically confirmed by both a neurosurgeon and neuroradiologist from an angiogram or magnetic resonance angiogram. Family members of included patients were also studied when possible.Patients and participating family members provided buccal swab samples (Isohelix SK-2S DNA buccal swab kits), medical records, neuroimaging studies, operative reports, and phenotypic data.
Ethics oversight	All procedures in this study comply with Yale University's Human Investigation Committee (HIC) and are approved by Yale University's Human Research Protection Program. Written informed consent was obtained from all adult participants. Written authorization was obtained from a parent or legal guardian for sample collection from all minors in this study.

Note that full information on the approval of the study protocol must also be provided in the manuscript.

Field-specific reporting

Please select the one below that is the best fit for your research. If you are not sure, read the appropriate sections before making your selection.

☒ Life sciences ☐ Behavioural & social sciences ☐ Ecological, evolutionary & environmental sciences

For a reference copy of the document with all sections, see nature.com/documents/nr-reporting-summary-flat.pdf

Life sciences study design

All studies must disclose on these points even when the disclosure is negative.

Sample size	We incorporated all samples that had undergone WES and satisfied our selection criteria at the data freeze point to bolster our statistical power for gene discovery. This cohort represents the largest group to date with well-defined mural or choroidal VOGMs, as confirmed by both a neurosurgeon and a neuroradiologist through angiogram or magnetic resonance angiogram.
Data exclusions	Patients were excluded if VOGM could not be confirmed or another cerebrovascular abnormality was identified during radiographic imaging review.
Replication	There was no replication of patient data because no other VOGM WES cohort was accessible to us at the time of data freeze.
Randomization	No randomization was performed to allocate samples into experimental groups as our study used a robust statistical framework designed to identify enrichment of rare mutations in genes by comparing observed number of mutations to the expectation independent of ethnic

background, sex, and consanguinity. Case cohort consists patients with VOGM. Control cohort consists of unaffected sibling or parent of proband with autism; ascertained for absence of autism.

Blinding

No blinding of patient data was needed given that all patients enter the study with unknown genetic status. Phenotypic clustering analysis is an unbiased algorithmic process and any further groupings of patients for integrative genomic analysis is based on predetermined numerical cutoffs generated by DenovolyzeR and the variant annotation software we employed.

Reporting for specific materials, systems and methods

We require information from authors about some types of materials, experimental systems and methods used in many studies. Here, indicate whether each material, system or method listed is relevant to your study. If you are not sure if a list item applies to your research, read the appropriate section before selecting a response.

Materials & experimental systems

n/a	Involved in the study
☐	☒ Antibodies
☐	☒ Eukaryotic cell lines
☒	☐ Palaeontology and archaeology
☐	☒ Animals and other organisms
☐	☒ Clinical data
☒	☐ Dual use research of concern

Methods

n/a	Involved in the study
☒	☐ ChIP-seq
☐	☒ Flow cytometry
☐	☒ MRI-based neuroimaging

Antibodies

Antibodies used

The following antibodies were used: For western blots: goat anti-EPHB4 polyclonal IgG (R&D systems, AF446), mouse anti-phosphotyrosine monoclonal IgG (clone 4Gl0, MilliporeSigma, 50-321), rabbit anti-ACTB polyclonal IgG (Cell Signaling Technologies, 4967), rabbit anti-TUBB monoclonal IgG (clone 9F3, Cell Signaling Technologies, 2128), and mouse anti-Myc monoclonal IgG (clone 9El0; EMD MilliporeSigma, OP10-200UG), goat anti-rabbit IgG-HRP (Cell Signaling Technology, 7074), horse anti-mouse IgG-HRP (Cell Signaling Technology, 7076), donkey antigoat IgG-HRP (Jackson Immunoresearch, 705-035-147). For whole mount: rat anti-CD31 antibody (clone MEC13.3, BD Biosciences, 550274), donkey antirat Alexa Fluor 488 antibody (Invitrogen, A21208). For immunofluorescence: rat anti-CD31 antibody (Dianova, SZ31), rabbit anti-LYVE1 IgG (Abcam, 14917), rabbit anti-active Caspase-3 IgG (AF835, R&D Systems), goat anti-Type IV Collagen (1340-01, Southern Biotech), donkey anti-rat F(ab)2 fragment Alexa Fluor 594 (Jackson Immunoresearch, 712-586-153), donkey anti-rabbit F(ab)2 fragment Alexa Fluor 488 (Jackson Immunoresearch, 711-546-152), donkey anti-goat F(ab)2 fragment Alexa Fluor 647 (Jackson Immunoresearch, 705-606-147).

Validation

Validation statements on the manufacturer's website:
anti-EPHB4: https://www.rndsystems.com/products/mouse-ephb4-antibody_af446; anti-phosphotyrosine: https://www.sigmaaldrich.com/US/en/, product/mm/05321; anti-ACTB: https://www.cellsignal.com/products/primary-antibodies/b-actin-antibody/4967; anti-TUBB: https://www.cellsignal.com/products/primary-antibodies/b-tubulin-9f3-rabbit-mab/2l28; anti-Myc: https://www.emdmillipore.com/US/en/product/Anti-c-Myc-Ab-1-Mouse-mAb-9E10,EMD_BIO-OP10?ReferrerURL=https%3A%2F%2Fwww.google.com%2F&bd=1; anti-rabbit IgG-HRP: https://www.cellsignal.com/products/secondary-anti bodies/anti-rabbit-igg-hrp-linked-antibody/7074; anti-mouse IgG-HRP:https://www.cellsignal.com/products/secondary-antibodies/antimouse-igg-hrp-linked-antibody/7076; anti-goat IgG-HRP: https://www.jacksonimmuno.com/catalog/products/705-035-147; anti-CD31: https://www.bdbiosciences.com/en-ca/products/reagents/flow-cytometry-reagents/research-reagents/single-colorantibodies-ruo/purifiedrat-anti-mouse-cd3l.550274; anti-rat Alexa Fluor 488 https://www.thermofisher.com/antibody/product/Donkey-anti-Rat-IgG-H-L-Highly-Cross-Adsorbed-Secondary-Antibody-Polyclonal/A-21208; anti-CD31: https://www.dianova.com/en/shop/dia-310-anti-cd3lmssw-from-rat-sz3l-unconj-for-mouse-ffpe-tissue; anti-LYVE1: https://www.abcam.com/products/primaryantibodies/lyve1-antibody-bsa-and-azide-free-ab14917.html; anti-Type IV Collagen: https://www.southernbiotech.com/goat-anti-type-iv-collagenunlb-1340-01; anti-Active Caspase 3: https://www.rndsystems.com/products/human-mouse-active-caspase-3-antibody_af835, donkey anti-goat F(ab)2 fragment Alexa fluor 647: https://www.jacksonimmuno.com/catalog/products/705-606-147; donkey anti-rabbit F(ab)2 fragment Alexa fluor 488: https://www.jacksonimmuno.com/catalog/products/711-546-152; donkey anti-rat F(ab)2 fragment Alexa fluor 594: https://www.jacksonimmuno.com/catalog/products/712-586-153.

Eukaryotic cell lines

Policy information about cell lines and Sex and Gender in Research

Cell line source(s)

Only Cos-7 cells (ATCC CRL-1651) been used in this study. Cells were purchased fresh from ATCC and were only passaged once or twice before use.

Authentication

Cells were purchased fresh from ATCC and were only passaged once or twice before use. See manufacturer's website for more information: https://www.atcc.org/products/ crl-165. We checked the morphology of the purchased COS-7 cells with the reported fibroblast-like characteristics and found them identical.

Mycoplasma contamination

Mycoplasma was not detected.

Commonly misidentified lines (See ICLAC register)	During these experiments, no commonly misidentified cell lines were used.

Animals and other research organisms

Policy information about studies involving animals; ARRIVE guidelines recommended for reporting animal research, and Sex and Gender in Research

Laboratory animals	All fish were obtained from the Zebrafish International Research Center (ZIRC). Adult Tg(kdr:gfp)zn1 zebrafish (AB background) were maintained in 3.0 L tanks with constant water flow (Iwaki Aquatic) under a 14h/10h light/dark cycle. They were fed GEMMA Micro 500 (Skretting USA) dry food twice daily supplemented with hatched Artemia once daily. Embryos were obtained via natural matings; these embryos were maintained at 28.5º C in E3 medium treated with 1-phenyl 2-thioruea (PTU, to prevent melanization for imaging) at a final concentration of 100 µM. All fish studied were between 2 and 3 days post fertilization.<br><br>Cdh5Ert2Cre mice were obtained from Cancer Research UK. Mice were kept on a C57BL/6J background (strain# 000664) from the Jackson laboratory. Experiments were performed on post coitus embryonal stage E9.5, E10.5, E17.5 and E18.5. sgRNA/Cas9 RNP and the template oligo were electroporated into C57Bl/6J (JAX) zygotes. Embryos were transferred to the oviducts of pseudopregnant CD-1 foster females using standard techniques (Nagy et al. 2003). Genotype screening of tissue biopsies from founder pups was performed by PCR amplification and Sanger sequencing, followed by breeding to establish germline transmission of the correctly targeted F867L allele. Mice were kept on a 12-hour light/dark cycle at a constant room temperature of 22°C ± 1°C and relative humidity of 40% ± 10%.
Wild animals	No wild animals were used in this study
Reporting on sex	Data from both male and female mice was collected, but no differences were observed.
Field-collected samples	Field collected samples were not involved in this study
Ethics oversight	All zebrafish care and handling was performed in accordance with Yale IACUC protocol 2019-20274; all mouse experiments complied with University of Michigan guidelines and were approved by the university of Michigan IACUC. Protocol number PRO00009847.

Note that full information on the approval of the study protocol must also be provided in the manuscript.

Clinical data

Policy information about clinical studies

All manuscripts should comply with the ICMJE guidelines for publication of clinical research and a completed CONSORT checklist must be included with all submissions.

Clinical trial registration	N/A. This study does not meet the definition of 'Clinical Trial' as defined by the National Institutes of Health or the World Health Organization. It is not currently registered at ClinicalTrials.gov
Study protocol	The study protocol is not publicly available, but is available upon request.
Data collection	Study participant data was collected via self administered survey as well as mined from medical records released to authors after HIPPA compliant medical release forms were provided by the study participants. Recruitment and data collection took place from January 2016 through August 2021.
Outcomes	N/A

Flow Cytometry

Plots

Confirm that:

☒ The axis labels state the marker and fluorochrome used (e.g. CD4-FITC).

☒ The axis scales are clearly visible. Include numbers along axes only for bottom left plot of group (a 'group' is an analysis of identical markers).

☒ All plots are contour plots with outliers or pseudocolor plots.

☒ A numerical value for number of cells or percentage (with statistics) is provided.

Methodology

Sample preparation	c-myc-tagged Ephb4 cDNA in pCMV6 was from Origene. Single K650N, R838W and F867L Ephb4 mutations were introduced by site-directed mutagenesis using a QuikChange II XL Site-Directed Mutagenesis Kit (Agilent) according to manufacturer's instructions. Cos-7 cells (ATCC) were cultured in DMEM supplemented with 10% FBS and 100 U/ml penicillin/streptomycin (all Thermo Fisher Scientific) in 10 cm culture dishes. At 60% confluency, cells were transfected with 10 mg of plasmid using Lipofectamine in Opti-MEM (both Thermo Fisher Scientific). To control for transfection efficiency, cells were co-transfected with 1 µg of pEGFP-N1 plasmid (Takara Bio USA). Where indicated, protein stability was assessed by adding 5 mg/ml

	cycloheximide (Sigma-Aldrich) to the cell growth medium for the indicated times prior to harvest. Cells were harvested 48 hours after transfection and washed 2 times with ice-cold PBS.
Instrument	GFP content was assessed by flow cytometry using BD Fortessa or BD FACSCanto instruments (BD Biosciences)
Software	FlowJo 10.7.1
Cell population abundance	Cell population abundance is not relevant to this study.
Gating strategy	Gating strategy is not relevant to this study.

☐ Tick this box to confirm that a figure exemplifying the gating strategy is provided in the Supplementary Information.

Magnetic resonance imaging

Experimental design

Design type	No task in our experimental design. Available MRI brain scans for any study participants who underwent whole exome sequencing were obtained and reviewed by an ABNS board certified neurosurgeon and board certified neuroradiologist to confirm the diagnosis of VOGM.
Design specifications	All available MRI brain scans were reviewed (from birth to time of entry in the study).
Behavioral performance measures	There were no behavioral performance measures in this study.

Acquisition

Imaging type(s)	Structural scans only
Field strength	1.5T and 3T
Sequence & imaging parameters	Clinical imaging protocols varied depending on institution but were within the "standard" for structural MRI brain scans. Sequences evaluated included (when available) 3D T1 weighted gradient recalled echo sequences (pre- and postcontrast), 2D T2 spin echo sequences, 2D T2 fluid attenuated inversion recovery sequences, all of which were of whole brain. All orientations were evaluated (axial, coronal sagittal) when available.
Area of acquisition	Whole brain scans

Diffusion MRI ☐ Used ☒ Not used

Preprocessing

Preprocessing software	N/A; processing was performed by clinical institution
Normalization	N/A; normalization was performed by clinical institution
Normalization template	N/A; normalization was performed by clinical institution
Noise and artifact removal	N/A; noise and artifact removal was performed by clinical institution
Volume censoring	N/A; volume censoring was performed by clinical institution

Statistical modeling & inference

Model type and settings	No statistical modeling was performed
Effect(s) tested	N/A

Specify type of analysis: ☒ Whole brain ☐ ROI-based ☐ Both

Statistic type for inference (See Eklund et al. 2016)	No statistical analysis on MRI images
Correction	N/A

Models & analysis

n/a	Involved in the study
☒	☐ Functional and/or effective connectivity
☒	☐ Graph analysis
☒	☐ Multivariate modeling or predictive analysis
